# Introducing 2‑Thioxocoumarins as a New Class of Photosensitizers for Antimicrobial Photodynamic Therapy

**DOI:** 10.1021/acsomega.6c07980

**Published:** 2026-10-02

**Authors:** Javier Ordóñez-Hernández, Abel Jaume-Masferrer, Jordi Hernando, Marc Martínez-Miguel, Claire Brazzi, Elies Molins, Rosario Núñez

**Affiliations:** † 54449 Institut de Ciència de Materials de Barcelona (ICMAB-CSIC), Carrer dels Til·lers, Cerdanyola del Vallès 08193, Spain; ‡ Departament de Química, Universitat Autònoma de Barcelona (UAB), Bellaterra (Cerdanyola del Vallès), Barcelona 08193, Spain

## Abstract

Antimicrobial photodynamic therapy (aPDT) has gained increasing attention as an alternative approach to combat bacterial resistance, although the development of efficient visible-light photosensitizers (PSs) remains a major challenge. Despite the extensive investigation of coumarin derivatives for photodynamic applications, 2-thioxocoumarins have not previously been explored for aPDT. Herein, we introduce 2-thioxocoumarins as a new class of photosensitizers for aPDT through the rational design and synthesis of four derivatives (**TCM-1**–**TCM-4**). Thionation, combined with strategic bromination, modulated the photophysical and photochemical properties of the coumarin scaffold, promoting visible-light absorption and efficient generation of reactive oxygen species (ROS) via both Type I and Type II photochemical pathways. Biological evaluation identified the monobrominated derivative **TCM-2** as the lead photosensitizer, exhibiting potent light-induced antibacterial activity against methicillin-sensitive and methicillin-resistant *Staphylococcus aureus* with minimum inhibitory concentrations of 4–8 μM and no detectable dark toxicity. Although the tribrominated derivatives (**TCM-3**–**TCM-4**) exhibited enhanced Type I ROS generation, this was not accompanied by a proportional increase in photodynamic antibacterial activity, highlighting that efficient photodynamic inactivation depends not only on ROS generation but also on molecular properties governing bacterial interactions. These findings establish 2-thioxocoumarins as promising antimicrobial PSs for surface disinfection in healthcare settings and as a valuable molecular scaffold for the development of next-generation antimicrobial photosensitizers.

## Graphical abstract

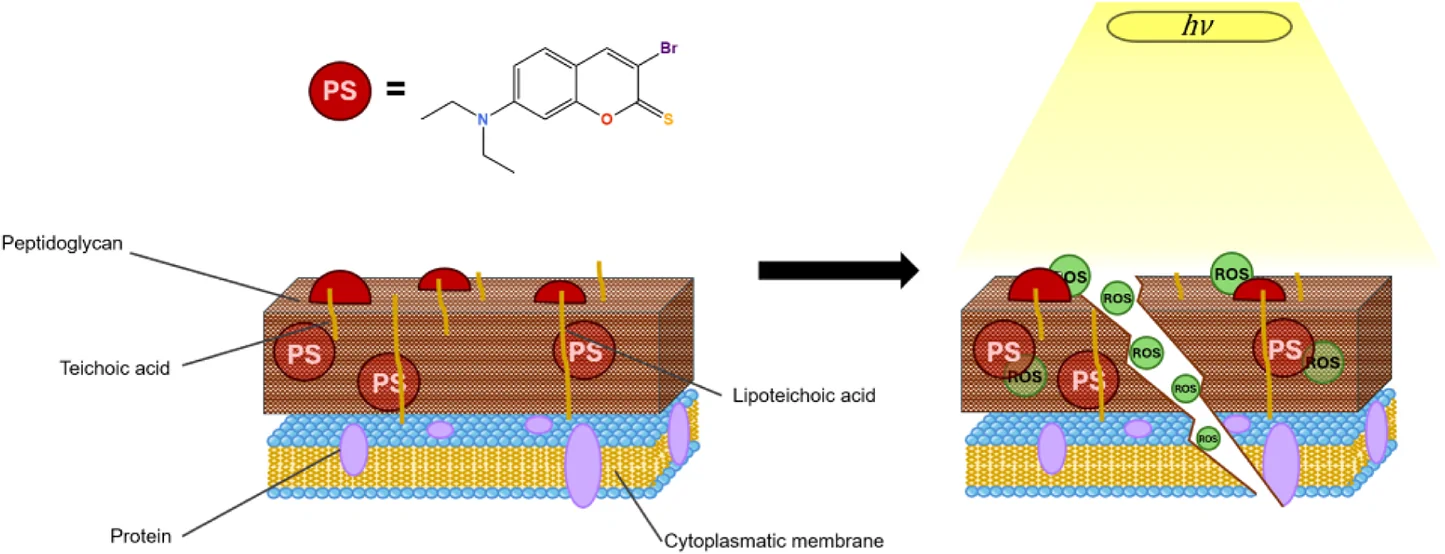

## 1 Introduction

The rapid emergence of microbial resistance to conventional antibiotics and antiseptics represents a critical and escalating challenge across medicine, chemistry, and the life sciences.^1^ According to reports from the European Centre for Disease Prevention and Control (ECDC), infections acquired in healthcare environments, particularly those involving multidrug-resistant (MDR) pathogens, represent a major concern for public health worldwide.^2–7^ These reports highlight the urgent need to develop therapeutic strategies that operate through mechanisms fundamentally distinct from those of traditional antimicrobial agents.

Among the alternative approaches currently under investigation, antimicrobial photodynamic therapy (aPDT) offers a promising option for the decontamination of healthcare-associated surfaces, including medical equipment, frequently touched surfaces, and implantable devices.^8–10^ In contrast to conventional antibiotics, aPDT offers several notable advantages, including reduced treatment times, minimal invasiveness, broad-spectrum activity independent of preexisting antibiotic resistance, and a markedly low propensity to induce resistance, even after repeated applications.^11–16^

This strategy relies on a photosensitizer (PS), a light-activatable compound that generates reactive oxygen species (ROS) upon visible-light irradiation. In particular, singlet oxygen (^1^O_2_) and other oxidative species induce irreversible damage to essential biomolecular components within microbial cells.^17,18^ Upon light absorption, the PS is promoted to its first excited singlet state (S_1_) from the ground state (S_0_), followed by possible intersystem crossing (ISC) to populate the longer-lived triplet state (T_1_). From this state, the PS can then initiate the production of ROS through two principal pathways:^19,20^ (i) Type I mechanism, in which electron- or hydrogen-transfer reactions give rise to radical species including superoxide (O_2_^•–^) and hydroxyl radicals (·OH); (ii) Type II mechanism, where the excited PS transfers energy to molecular oxygen, resulting in ^1^O_2_. Both pathways ultimately induce oxidative damage in biological systems. These processes may occur concurrently, although the Type II pathway is often dominant under normoxic conditions due to its higher efficiency.^21,22^

At present, most conventional PSs primarily generate ^1^O_2_ via the Type II pathway,^23^ including systems based on BODIPY and rhodamine scaffolds.^24–27^ However, the effectiveness of this mechanism is highly dependent on oxygen availability, thereby limiting its applicability in hypoxic environments such as solid tumors or certain bacterial infections.^28,29^ In contrast, Type I PSs are less dependent on oxygen concentration and can generate cytotoxic radical species under oxygen-deficient conditions, thereby partially overcoming hypoxia-related limitations.^30,31^ In particular, superoxide radicals can undergo further transformation, either enzymatically (e.g., via superoxide dismutase) or through chemical pathways, to produce highly reactive ·OH, enhancing overall oxidative stress.

A common limitation of traditional PSs is their relatively low triplet-state quantum yield, which reduces the efficiency of ROS generation. To address this issue, structural modifications that increase spin-orbit coupling are often employed to promote ISC.^32^ In this context, thiocarbonyl groups have attracted considerable attention for their ability to favor triplet-state formation and enhance photodynamic performance.^33–35^ Similarly, incorporation of heavy halogen atoms, particularly bromine or iodine, can effectively facilitate ISC, as demonstrated in halogenated PSs based on BODIPY cores.^36,37^

Against this background, coumarin-based scaffolds have emerged as versatile molecular frameworks for PS design, owing to their attractive photophysical properties, structural tunability, and synthetic accessibility.^38–40^ In particular, the incorporation of thiocarbonyl functionalities and heavy halogen atoms into coumarin derivatives represents a promising strategy to enhance ISC and promote efficient population of the triplet state.^41,42^ These structural modifications are expected to improve overall ROS generation by enabling both Type I and Type II photochemical pathways, thereby increasing the versatility and effectiveness of the PS under varying oxygen conditions.^43,44^ Notably, although coumarin-based PSs have been widely investigated, the application of 2-thioxocoumarin derivatives in aPDT remains largely unexplored,^45^ representing a rational approach to developing next-generation PSs for this application.

To our knowledge, 2-thioxocoumarins have not previously been reported as photosensitizers for aPDT. Herein, a series of 2-thioxocoumarins (**TCM-1**–**TCM-4**, Chart [Fig chart1]) was rationally designed and synthesized to investigate how sulfur incorporation and bromination influence their photophysical properties, ROS generation, and biological activity. Replacing the lactone oxygen with sulfur, together with strategic bromination, was hypothesized to red-shift the absorption profile, enhance ISC , and promote efficient ROS generation via Type I and Type II photochemical mechanisms. The resulting compounds were systematically investigated through comprehensive structural and photophysical characterization, single-crystal X-ray diffraction analysis, ROS generation studies, antibacterial evaluation against Gram-positive and Gram-negative bacteria, and phototoxicity studies in human fibroblasts. Among the series, **TCM-2** emerged as the lead compound, exhibiting potent light-induced antibacterial activity against methicillin-sensitive *Staphylococcus aureus* (MSSA) and methicillin-resistant *Staphylococcus aureus* (MRSA). These findings highlight 2-thioxocoumarins as promising antimicrobial PSs for surface disinfection in healthcare settings and establish this set of compounds as a valuable molecular framework for designing new antimicrobial PSs with improved antibacterial efficacy and wider applicability in aPDT.

**1 chart1:**
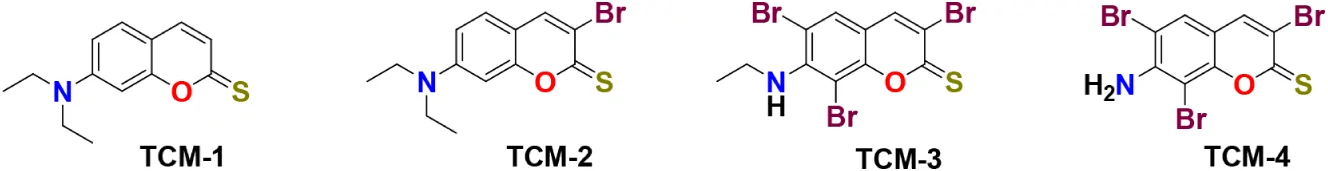
Structures of PSs Based on 2-Thioxocoumarins

## 2 Results and Discussion

### 2.1 Design, Synthesis, and Structural Characterization

2-Thioxocoumarins displayed in Chart [Fig chart1] were synthesized following the synthetic route depicted in Scheme 1. The coumarin core was initially prepared by Knoevenagel condensation of 4-(diethylamino)­salicylaldehyde with diethyl malonate in absolute ethanol under reflux, using piperidine as the base. Subsequent hydrolysis and decarboxylation under strongly acidic conditions (concentrated hydrochloric acid/glacial acetic acid) afforded compound **CM-1**. Selective bromination at the 3-position of **CM-1** using 1.1 equiv of bromine in acetic acid provided **CM-2** in good yield. In contrast, treatment of **CM-1** with 13 equiv of bromine resulted in polybrominated derivatives **CM-3** and **CM-4**, both bearing bromine substituents at the 3, 6, and 8 positions of the coumarin framework.

Notably, during the polybromination process, partial and complete *N*-dealkylation were observed. Compound **CM-3** underwent the loss of one ethyl group, whereas **CM-4** lost both ethyl substituents, leading to the formation of a secondary amine in **CM-3** and a primary amine in **CM-4**. These transformations likely proceed via an S_N_2 mechanism involving the corresponding alkylammonium intermediate and bromide ion. The four 2-thioxocoumarins (**TCM-1**–**TCM-4**) were synthesized from their corresponding parent coumarins in moderate 40–52% yields through thionation of the lactone carbonyl group using Lawesson’s reagent.

All compounds were characterized by elemental analysis, IR-ATR, ^1^H and ^13^C NMR spectroscopy (Figures S1–S16), UV–Vis absorption and fluorescence emission spectroscopy. For all 2-thioxocoumarin-based PSs, the IR-ATR spectra display strong ν­(C=S) absorption bands at 1070–1079 cm^–1^, consistent with the presence of the thiocarbonyl group within the 2-thioxocoumarin scaffold.

The ^1^H NMR spectrum of **TCM-1** (Figure S9) displays three doublets at δ 7.29, 7.28, and 6.95 ppm, assigned to H-4, H-5, and H-3, respectively. The remaining aromatic protons, H-6 and H-8, appear as a multiplet at δ 6.70–6.64 ppm. In the aliphatic region, a quartet at δ 3.46 ppm and a triplet at δ 1.25 ppm are assigned to the CH_2_ (H-9/H-9′) and CH_3_ (H-10/H-10′) protons of the diethylamino group. For **TCM-2**, the ^1^H NMR spectrum (Figure S11) shows a singlet at δ 7.84 ppm corresponding to H-4. The change in multiplicity and the downfield shift relative to H-4 in **TCM-1** are consistent with the electron-withdrawing influence of the bromine substituent at C-3. The remaining signals retain the same multiplicities and exhibit very similar chemical shifts to those observed for **TCM-1**.

**Scheme 1:**
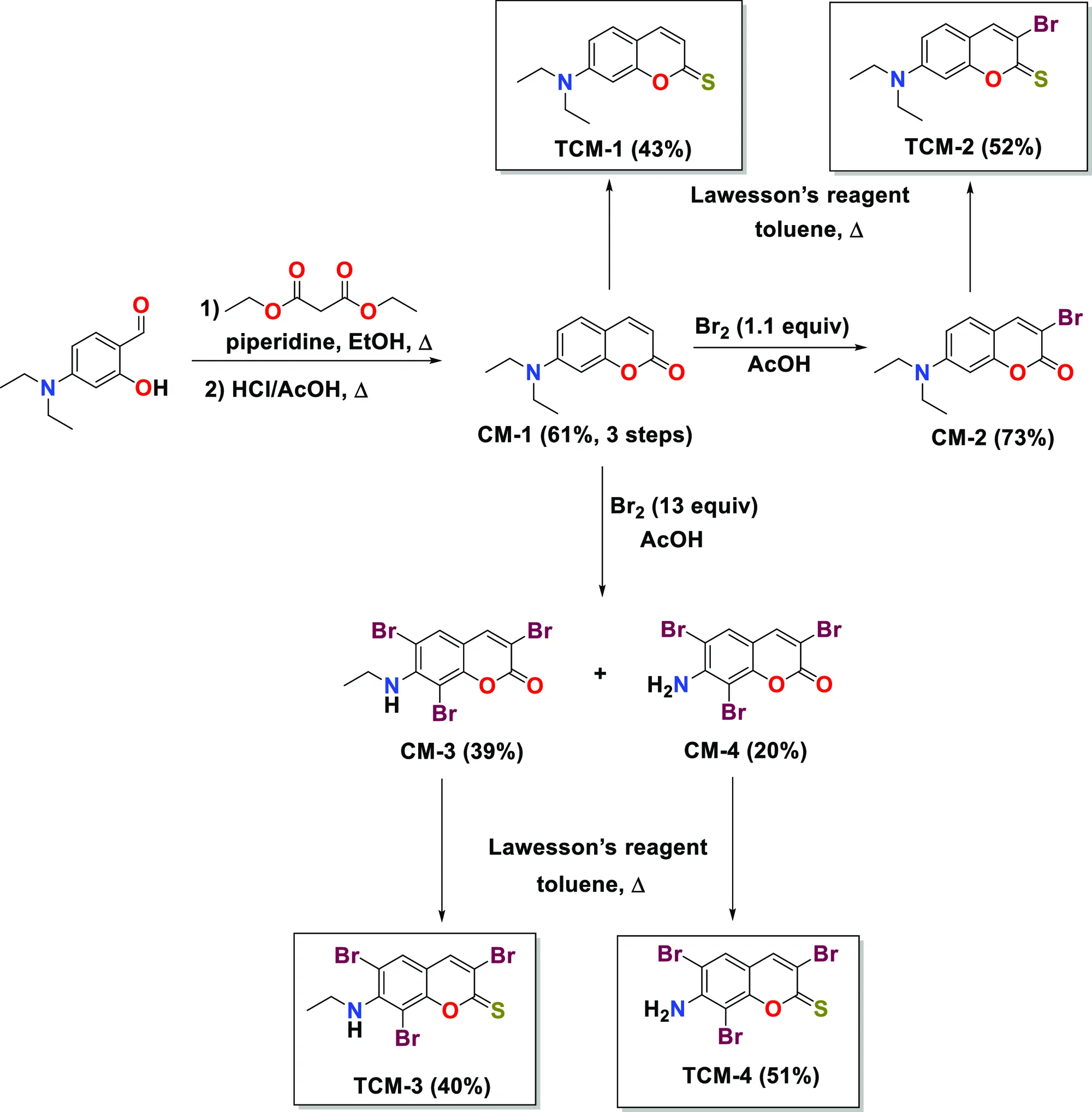
Synthetic Route Used to Prepare the PSs

The ^1^H NMR spectra of the tribrominated 2-thioxocoumarins **TCM-3** (Figure S13) and **TCM-4** (Figure S15) display a simplified aromatic pattern, with only two singlets assigned to H-4 and H-5 at δ 8.44 and 8.02 ppm for **TCM-3**, and at δ 8.37 and 7.93 ppm for **TCM-4**. In addition, a triplet at δ 5.67 ppm in **TCM-3** is assigned to the N–H proton of the ethylamino group, while the −NH_2_ protons in **TCM-4** appear as a broad singlet at δ 6.70 ppm. In the aliphatic region, the ethylamino group of **TCM-3** gives rise to a quintet at δ 3.64 ppm and a triplet at δ 1.18 ppm, corresponding to H-9 and H-10, respectively.

The ^13^C NMR spectra of **TCM-1**–**TCM-4** (Figures S10, S12, S14, S16) provided additional confirmation of thiocarbonyl formation. The signal assigned to the C=S carbon appears markedly downfield (δ 197.6–191.6 ppm) relative to the lactone C=O carbon in the corresponding parent coumarins (δ 162.0–157.1 ppm), consistent with the increased deshielding associated with the thiocarbonyl group and in agreement with reported literature values for thiocarbonyl carbons.^46^ This characteristic shift supports the successful conversion of the carbonyl into a thiocarbonyl functionality.

Additional characteristic signals in **TCM-1**–**TCM-4** that differ significantly from those of the parent coumarins correspond to carbons C-3, C-4, and C-4a (see Table 1). These carbons are located in close proximity to the C=S group and are therefore strongly influenced by its electronic effects.

**Table 1:** Selected ^13^C NMR Chemical Shifts (δ, ppm) for **CM-1**–**CM-4** and **TCM-1**–**TCM-4**

Compound	C-2 (C=S)	C-3	C-4	C-4a
**CM-1**	162.0	108.8	143.6	108.0
**TCM-1**	197.6	123.0	136.1	110.6
**CM-2**	158.3	102.8	144.7	108.8
**TCM-2**	192.1	110.9	138.3	111.0
**CM-3**	157.1	107.9	144.8	114.6
**TCM-3**	191.4	110.5	137.8	120.5
**CM-4**	157.2	105.4	145.4	111.8
**TCM-4**	191.6	106.3	138.9	119.2

Diffraction-quality crystals of **TCM-2**, **TCM-3**, and **TCM-4** were obtained by slow evaporation of chloroform. Single-crystal X-ray diffraction analysis confirmed the molecular structures of these compounds. **TCM-2** crystallized in the monoclinic P21/n space group (Figure 1), exhibiting a nearly planar conformation, including the terminal aliphatic substituent. Molecules are arranged as zig-zag chains connected through S···Br contacts (d = 3.458 Å). Chains are coupled, forming 2D layers via CH···S and π–π interactions, and these layers are stabilized by van der Waals interactions arising from face-to-face methyl groups. **TCM-3** crystallized in the monoclinic P21/n space group (Figure 1), adopting a nearly planar conformation. Molecules are arranged as zig-zag chains connected through S···Br contacts (d = 3.661 Å). Chains are mainly coupled by π-π interactions, resulting in a 3D arrangement. **TCM-4** crystallized in the monoclinic P21/c space group (Figure 1), showing an expected planar conformation. Molecules are arranged as pairs through two symmetrical S···Br contacts (d = 3.566 Å), which form almost planar sheets through CH···S intermolecular interactions (C···S distance 3.446 Å). These sheets are packed along the c-axis through π–π interactions. Complete crystallographic data, additional crystallographic parameters, and selected bond lengths and angles are provided in Tables S1–S15.

**Figure 1:**
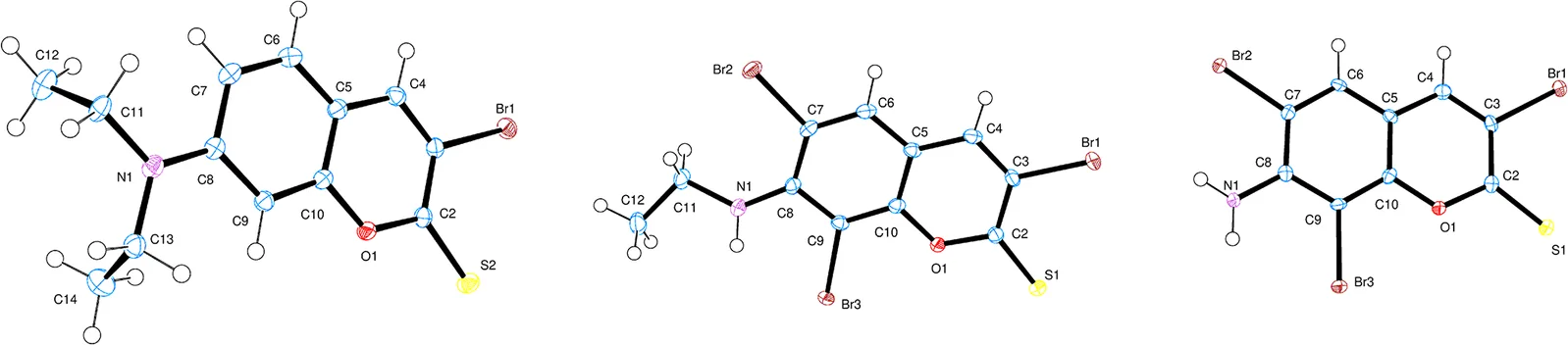
ORTEP representations of **TCM-2**, **TCM-3**, and **TCM-4**.

### 2.2 Photophysical and Photochemical Studies

The photophysical properties of all compounds were analyzed in acetonitrile (ACN) at room temperature using UV–Vis absorption and fluorescence emission spectroscopies. In Table 2, the data obtained for their maximum absorption and emission wavelengths, molar absorptivity coefficients (ε), fluorescence quantum yields (Φ_f_), and ROS generation are summarized.

**Table 2:** Photophysical Properties of Coumarin and 2-Thioxocoumarin Derivatives

Compound	*λ*_abs,max_ (nm)	*ε*_abs,max_ (M^–1^ cm^–1^)	*λ*_f,max_ (nm)	*Φ*_f_ ^a^	k_ROS_/Abs_405 nm_ ^b^ (s^–1^)	*Φ*_Δ_ ^c^
CM-1	372	33849	442	0.63	-	-
CM-2	391	29230	467	0.36	-	-
CM-3	359	26053	451	0.16	-	-
CM-4	356	22190	433	0.47	-	-
TCM-1	462	39181	n.d^d^	-	0.0004	0.18 ± 0.01
TCM-2	483	31778	n.d^d^	-	0.0007	0.34 ± 0.01
TCM-3	433	25479	n.d^d^	-	0.0047	0.33 ± 0.01
TCM-4	431	32708	n.d^d^	-	0.0040	0.36 ± 0.02

a Determined in ACN solution at *λ*_exc_ = 325 nm, relative to quinine sulfate (*Φ*_f_ = 0.54 in 0.1 M H_2_SO_4_).^52^

b Ratio between the rate constant of ROS generation determined by monoexponential fitting of DHR-123 data (k_ROS_) and the absorbance of the PS at 405 nm.

c Determined in ACN solution at *λ*_exc_ = 450 nm, using DMA as a ^1^O_2_ chemical probe, relative to perinaphthenone (PN) (*Φ*_Δ_ = 1 in ACN).^53^

d Not detectable under the experimental conditions. A dash (-) indicates not determined

As expected, coumarin derivatives showed strong absorption in the near-UV region, assigned to the lowest-energy π–π* electronic transition, with absorption maxima spanning 356–391 nm (Figure 2, Table 2). The introduction of electron-withdrawing bromine atoms induced a shift in this absorption band, which was contingent upon their number and positional arrangement. In **CM-2**, the incorporation of a bromine atom at position 3 enhanced the push-pull character of the coumarin chromophore, causing a red-shift of 19 nm of the absorption band relative to the parent **CM-1**. Conversely, the opposite effect was observed in compounds **CM-3** and **CM-4**, where the addition of two bromine atoms to the amino-substituted aryl ring reduced the push-pull character of the coumarin chromophore, leading to hypsochromic shifts of 13 and 16 nm in the absorption band, respectively, relative to **CM-1**. Additionally, the molar absorptivity coefficient decreased upon bromination, with reductions of 14%, 23%, and 34% for **CM-2**, **CM-3,** and **CM-4**, respectively, compared to **CM-1**.

**Figure 2:**
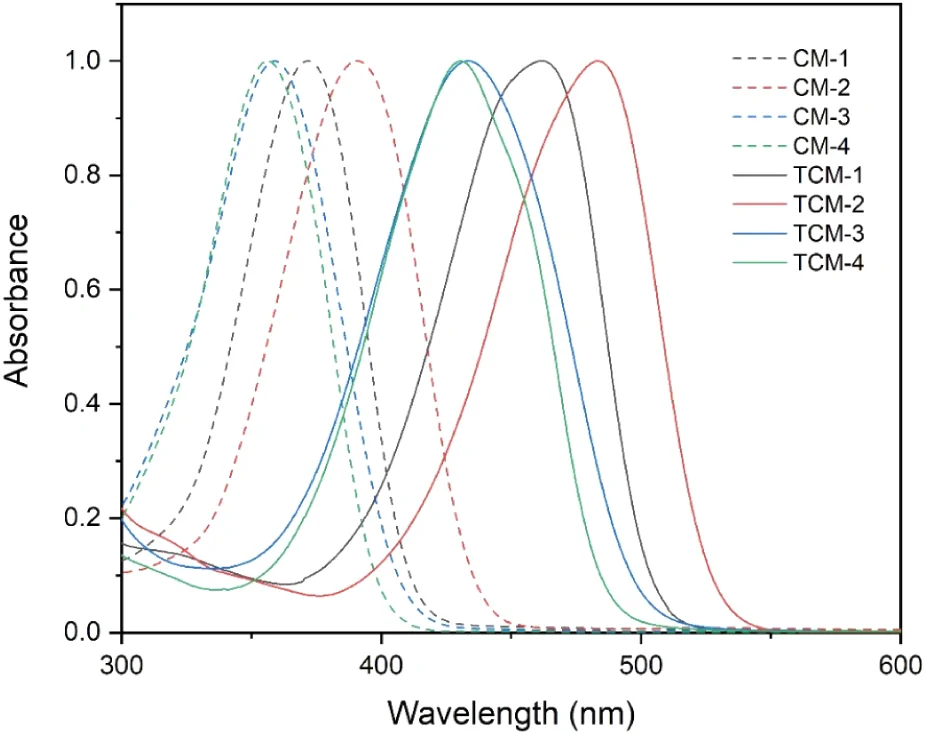
Absorption spectra of all compounds in ACN, normalized to their respective maxima.

Notably, 2-thioxocoumarin derivatives showed red-shifted absorption spectra with respect to their homologous coumarins, exhibiting absorption maxima in the range 431–483 nm. In particular, replacing the oxygen atom at position 2 of the chromenone core with sulfur bathochromically shifted the absorption maxima by approximately 90 nm for **TCM-1** and **TCM-2** and by about 75 nm for compounds **TCM-3** and **TCM-4** (Figure 2, Table 2). These compounds presented similar bromination effects on their absorption properties, registering bathochromic (**TCM-2**, 21 nm) or hypsochromic shifts (**TCM-3**, 29 nm; **TCM-4**, 31 nm) as well as a decrease in the molar absorptivity coefficient (18% for **TCM-2**, 35% for **TCM-3**, and 16% for **TCM-4**) when compared to **TCM-1**.

Analysis of the emission properties revealed more pronounced differences among the compounds in ACN (Figure S17 and Table 2). While **CM-1** showed bright fluorescence at λ_f,max_ = 442 nm with high quantum yield (*Φ*_f_ = 0.63), bromination of its coumarin core significantly reduced emission efficiency: *Φ*_f_ = 0.36, 0.16, and 0.47 for **CM-2**, **CM-3,** and **CM-4**, respectively. This effect can be ascribed to the heavy-atom effect, where increased spin-orbit coupling facilitates the population of non-emissive triplet states through ISC.^48^ However, a much more dramatic effect on fluorescence was observed for 2-thioxocoumarins **TCM-1**–**TCM-4**. Irrespective of the number of bromine atoms introduced, all these compounds showed complete emission quenching, which is typically attributed to the changes induced in the electronic structure of the chromophore by thionation.^49–51^ Introduction of a thiocarbonyl group in these molecules stabilizes nonradiative relaxation pathways, especially by promoting population of triplet states through the heavy-atom contribution of sulfur and the interaction between nπ* and ππ* states.^50,51^ As a consequence, 2-thioxocoumarins show two advantageous properties compared with coumarins for photodynamic therapy (PDT) applications: they exhibit strongly red-shifted absorption and efficient triplet-state generation.

In light of that, we investigated the ability of **TCM-1**–**TCM-4** to generate ROS through both Type I and Type II mechanisms. To evaluate the ROS-generating capacity associated with Type I photochemical pathways, dihydrorhodamine 123 (DHR-123) was employed as a probe, although it lacks specificity for individual radical species such as O_2_^•–^ and ·OH.^54^ The ROS-induced photooxidation of DHR-123 was investigated by irradiating mixtures of this probe and each of the PSs under violet-blue light (λ_exc_ = 405 nm), and monitoring the corresponding absorption changes over time. Figures 3a and S18–S21 show the progressive emergence of a new absorption band centered at approximately 500 nm in all cases upon irradiation, which is attributed to the formation of oxidized rhodamine 123 (Rh-123). These results therefore indicate that all 2-thioxocoumarin derivatives (**TCM-1**–**TCM-4**) can generate ROS via Type I photochemical pathways.

**Figure 3:**
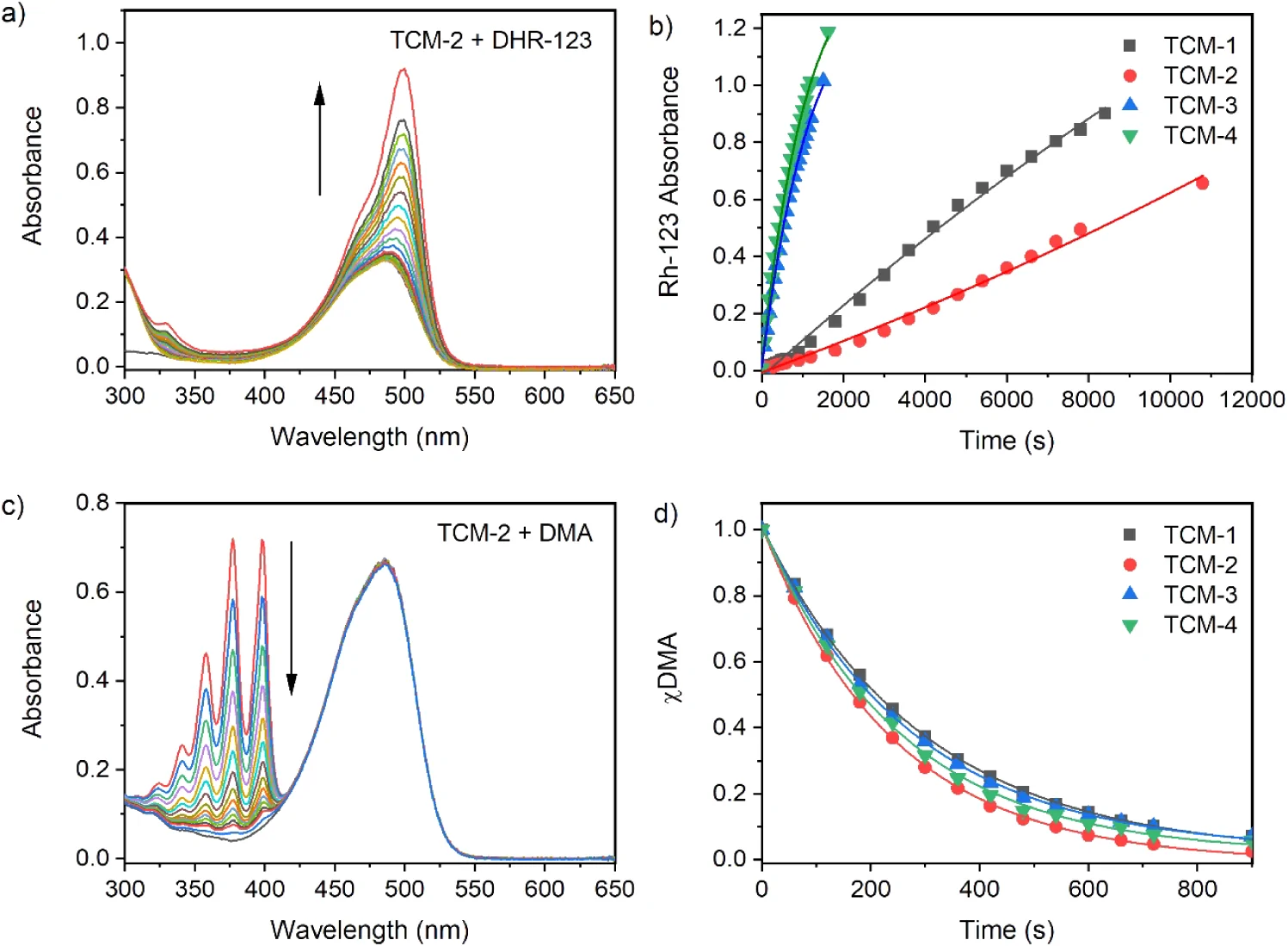
a) Variation in the absorption spectrum of a mixture containing **TCM-2** (*c* = 1.25 × 10^–5^ M) and DHR-123 (*c* = 3.75 × 10^–5^ M) in ACN upon irradiation using a violet-blue LED (λ_exc_ = 405 nm, power = 71 mW cm^–2^, irradiation from 0 to 10800 s). b) DHR-123 photooxidation kinetics obtained for mixtures containing DHR-123 (*c* = 3.75 × 10^–5^ M) and **TCM-1**–**TCM-4** (*c* = 1.25 × 10^–5^ M) in ACN. Symbols represent the experimental variation of the absorbance of rhodamine 123 (Rh-123) with the irradiation time for each mixture, while solid lines correspond to monoexponential fits of the experimental data. c) Variation in the absorption spectra of a mixture containing **TCM-2** (*c* = 2.5 × 10^–5^ M) and DMA (*c* = 7.5 × 10^–5^ M) in ACN upon irradiation with a cw blue laser (λ_exc_ = 450 nm, power = 80 mW cm^–2^, irradiation from 0 to 900 s). d) DMA photooxidation kinetics obtained for mixtures containing DMA (*c* = 7.5 × 10^–5^ M) and **TCM-1**–**TCM-4** (*c* = 2.5 × 10^–5^ M) in ACN. Symbols represent the experimental variation of the molar fraction of DMA (χ_DMA_) with the irradiation time for each mixture, while solid lines are fits to a monoexponential model.

A more quantitative analysis of Type I ROS generation by these compounds was obtained from the monoexponential fit of the DHR-123 photooxidation kinetics (Figure 3b). When the apparent rate constants retrieved from these fits were normalized with respect to the absorbance of each PS at 405 nm (k_ROS_/Abs_405 nm_, Table 2), significant differences in ROS generation efficiency were observed among the 2-thioxocoumarin derivatives. **TCM-3** and **TCM-4** exhibited markedly higher k_ROS_/Abs_405 nm_ values (0.0047 and 0.0040 s^–1^, respectively) compared to **TCM-2** (0.0007 s^–1^) and **TCM-1** (0.0004 s^–1^), indicating a substantially greater ability to promote photoinduced radical-mediated processes. As radical-type ROS are normally generated through electron-transfer processes involving PS triplet states, two main factors should account for this result. First, the larger number of bromine substituents in **TCM-3** and **TCM-4**, which should further enhance triplet-state formation due to heavy-atom effects.^32–37,48^ Second, the change in electronic distribution that these bromine atoms must cause in the triplet state of **TCM-3** and **TCM-4** with respect to the push-pull, monobrominated **TCM-2**, which seems to favor electron transfer processes toward oxygen for producing superoxide radical anions.

Based on these observations, a plausible Type I mechanism for these 2-thioxocoumarins involves photoexcitation followed by efficient ISC to a long-lived triplet excited state, as previously reported for thionated coumarins.^50^ The resulting triplet state is proposed to promote electron-transfer processes toward molecular oxygen, leading to the formation of O_2_^•–^, which may subsequently undergo secondary redox reactions to generate additional ROS (e.g., H_2_O_2_ and ·OH).^19^ Although the transient intermediates involved in this pathway were not directly characterized in the present study, this proposal is consistent with the Type I ROS generation detected using DHR-123.

^1^O_2_ generation (i.e., Type II ROS formation) was also evaluated for **TCM-1**–**TCM-4** in ACN (Table 2). This was assessed by monitoring the photooxidation kinetics of 9,10-dimethylanthracene (DMA), employed as a ^1^O_2_ chemical trap,^55,56^ in the presence of each PS under monochromatic blue-light irradiation (λ_exc_ = 450 nm). The decrease in DMA concentration over time was monitored by UV–Vis absorption measurements, enabling estimation of the ^1^O_2_ production rates for each compound (Figures 3c and S22–S25). The kinetic data were well described by a monoexponential model, from which apparent rate constants were extracted. These values were referenced against a standard (perinaphthenone, PN)^57^ under identical irradiation conditions to determine the singlet-oxygen quantum yields (*Φ*_Δ_) of the 2-thioxocoumarin derivatives. As shown in Table 2, **TCM-2**–**TCM-4** (*Φ*_Δ_ = 0.33–0.36) exhibited significantly higher ^1^O_2_ generation efficiencies compared to **TCM-1** (*Φ*_Δ_ = 0.18). This enhancement can be directly correlated with the incorporation of heavy bromine atoms into **TCM-2**–**TCM-4**, which is known to further favor ISC via increased spin-orbit coupling, thereby promoting more efficient population of the triplet excited state. As a result, these derivatives exhibit improved energy transfer to molecular oxygen, leading to enhanced ^1^O_2_ generation. The fact that similar *Φ*_Δ_ values were determined for tribrominated **TCM-3** and **TCM-4** relative to monobrominated **TCM-2** could be ascribed to the larger Type I ROS generation efficiency of the former, which should counterbalance the larger triplet-state population expected with increasing bromination degree.

Despite the partial and complete *N*-dealkylation observed for **TCM-3** and **TCM-4**, respectively, both derivatives showed remarkably similar photophysical properties, suggesting that this structural modification has only a minor influence on the photophysical behavior of these highly brominated 2-thioxocoumarins.

Taken together, these results demonstrate that the 2-thioxocoumarin derivatives can generate ROS via both Type I and Type II photochemical pathways. While the presence of the thiocarbonyl group enables ROS generation in all compounds, incorporating bromine atoms significantly enhances the generation of both radical species and ^1^O_2_ by promoting ISC. This dual photochemical behavior highlights the potential of halogenated 2-thioxocoumarins as versatile PSs, capable of operating under varying oxygen levels and thereby improving photodynamic performance.

### 2.3 Biological Activity

#### 2.3.1 Evaluation of Antimicrobial Properties

The antibacterial activity of the synthesized 2-thioxocoumarins (**TCM-1**–**TCM-4**) was evaluated to assess their potential as PSs for aPDT. Minimum inhibitory concentration (MIC) assays were performed against methicillin-sensitive *Staphylococcus aureus* (MSSA) and methicillin-resistant *Staphylococcus aureus* (MRSA), together with the Gram-negative strains *Escherichia coli* and *Pseudomonas aeruginosa*. All experiments were conducted both in the presence and absence of irradiation using a blue LED light source.

Upon illumination, antibacterial activity against Gram-positive planktonic bacteria was observed for **TCM-1**, **TCM-2**, and **TCM-4** (Table 3), with **TCM-2** showing the highest potency. In contrast, **TCM-3** did not exhibit any detectable antibacterial activity under the tested conditions, despite its demonstrated ability to generate ROS, as shown in the photochemical studies. Moreover, **TCM-3** precipitated after the 24 h incubation period, suggesting lower stability in the broth under the assay conditions. No growth inhibition was observed in the absence of light for any of the compounds, confirming that their antibacterial effects are strictly light-dependent. This absence of dark toxicity is a desirable feature for aPDT applications, as it minimizes undesired damage to surrounding biological systems.^58^

None of the compounds showed activity against Gram-negative bacteria under either irradiated or dark conditions (Table 3). This behavior can be rationalized by structural differences between the cell envelopes of Gram-positive and Gram-negative bacteria. Gram-negative bacteria possess an additional lipopolysaccharide-rich outer membrane that acts as an effective permeability barrier, restricting the penetration of many small molecules, particularly hydrophobic or bulky ones, while allowing the diffusion of small hydrophilic compounds through porin channels.^59^ As a result, the interaction of the 2-thioxocoumarin derivatives with intracellular targets is likely limited, preventing efficient photodynamic inactivation.

Among the active compounds, **TCM-2** displayed the most promising antibacterial activity, achieving MIC values of 4–8 μM against both MSSA and MRSA under irradiation, while **TCM-1** exhibited moderate activity and **TCM-4** had lower efficacy. Notably, despite its demonstrated ability to generate ROS through both Type I and Type II pathways, **TCM-3** remained inactive, highlighting that ROS generation alone is insufficient to confer biological activity. This behavior is likely associated with physicochemical factors such as increased steric bulk and reduced solubility, which may promote aggregation or precipitation in the biological medium, thereby limiting its effective concentration and interaction with bacterial cells.

**Table 3:** Minimum Inhibitory Concentration (MIC) of Each Tested Compound against an Initial Inoculum of ∼10^5^ CFU/mL^a^

	MIC (μM)							
	TCM-1		TCM-2		TCM-3		TCM-4	
Strain	Light	Dark	Light	Dark	Light	Dark	Light	Dark
*Gram-positive*								
*S. aureus*	16–32	>256	4–8	>256	>256	>256	32–64	>256
*S. aureus* *(methicillin resistant)*	32–64	>256	4–8	>256	>256	>256	32–64	>256
*Gram-negative*								
*E. coli*	>256	>256	>256	>256	>256	>256	>256	>256
*P. aeruginosa*	>256	>256	>256	>256	>256	>256	>256	>256

a The light dose was 25.7 J cm^–2^.

In terms of antibacterial activity against Gram-positive planktonic bacteria, **TCM-2** was the most active compound of the series, exhibiting MIC values of 4–8 μM against the tested*S. aureus* strains under a light dose of 25.7 J cm^–2^. For context, well-established PSs such as rose bengal and methylene blue have been reported to display MIC values of approximately 0.6 and 2 μM against MSSA, although under markedly different irradiation conditions, with light doses of 2.25 and 18 J cm^–2^, respectively.^58,60–63^ **TCM-1** showed lower antibacterial activity than **TCM-2**. Importantly, rose bengal and methylene blue have been reported to exhibit antibacterial activity under dark conditions at concentrations around 50 μM, whereas none of the compounds investigated in this work showed detectable antibacterial activity in the absence of light at the concentrations evaluated.

Due to their higher efficacy observed in previous MIC experiments, **TCM-1** and **TCM-2** were selected for further dose-response studies against Gram-positive strains.

**TCM-1** achieved a 3-log_10_ reduction of the initial inoculum at 64 μM upon illumination against MSSA, and at 128 μM no remaining bacteria were able to be detected by the assay (Figure 4a). In MRSA, the effect was milder, achieving a 1-log_10_ reduction at 128 μM (Figure 4b).

**Figure 4:**
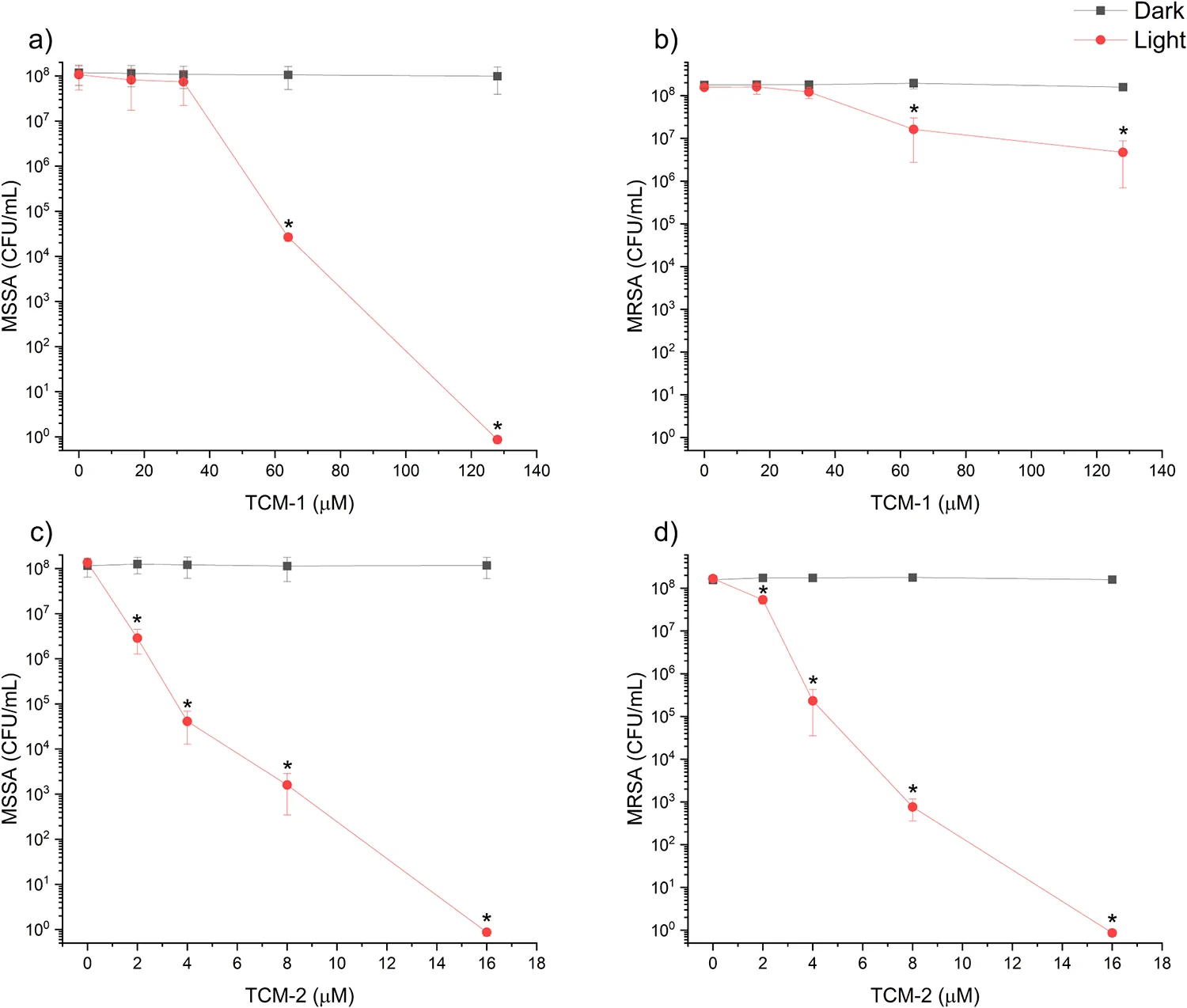
Dose-response curves for **TCM-1** against a) MSSA and b) MRSA, and **TCM-2** against c) MSSA and d) MRSA, kept in the dark (black lines) or exposed to the blue LED source (red lines), with an initial inoculum of ∼10^8^ CFU/mL. The light dose was 25.7 J cm^–2^. Data represent the mean of three biological replicates and the SD. Statistical significance: *p < 0.05.

In accordance with the previous MIC data, **TCM-2** showed the highest efficacy, achieving a 3-log_10_ reduction at 4 μM against MSSA (Figure 4c) and a 3-log_10_ reduction at 8 μM against MRSA (Figure 4d), with no remaining bacteria able to be detected at 16 μM in both strains.

These results indicate that although all compounds can generate ROS upon photoexcitation, their antibacterial efficacy is not solely determined by their intrinsic photophysical and photochemical properties. In particular, the marked difference between the high activity of **TCM-2** and the complete inactivity of **TCM-3**, despite comparable ROS-generating capability, highlights the critical role of physicochemical factors such as molecular size, solubility, and effective interaction with bacterial cells. This suggests that, beyond efficient ROS production, appropriate molecular properties governing bioavailability and cellular accessibility are essential for achieving effective photodynamic inactivation. In this context, the combined photochemical results indicate that both Type I and Type II mechanisms contribute to ROS generation in these systems; however, efficient antibacterial activity requires not only ROS production but also favorable molecular properties that enable effective interaction with bacterial cells.

#### 2.3.2 Evaluation of Light-Induced Cytotoxicity against Eukaryotic Cells

Following the antibacterial photodynamic evaluation, **TCM-1** and **TCM-2**, the two compounds exhibiting the most promising antibacterial activity under light, were selected for preliminary phototoxicity studies in a human skin fibroblast cell line (1BR.3.G). As both compounds require blue-light irradiation to generate ROS, and given the limited tissue penetration of blue light,^64^ this study was conceived as a proof-of-concept evaluation for potential topical photodynamic applications.

1BR.3.G cells were incubated with **TCM-1** or **TCM-2** over a concentration range of 100–0.01 μM and were either exposed to blue-light irradiation or maintained in the dark. Cell viability was subsequently determined using a resazurin assay. Results presented in Figure 5 indicate that both compounds exhibited a clear light-dependent cytotoxic response, whereas only slight dark toxicity was observed for **TCM-1** at the highest concentration tested (Figure 5a). Dose-response analysis of the irradiated samples afforded IC_50_ values of 3.05 and 0.95 μM for **TCM-1** and **TCM-2**, respectively. The greater phototoxicity of **TCM-2** (Figure 5b) further supports the superior photosensitizing properties demonstrated throughout this study, reinforcing its identification as the lead PS in this series. These findings further support the beneficial effects of sulfur incorporation and bromination on the photodynamic performance of coumarin-based PSs.

**Figure 5:**
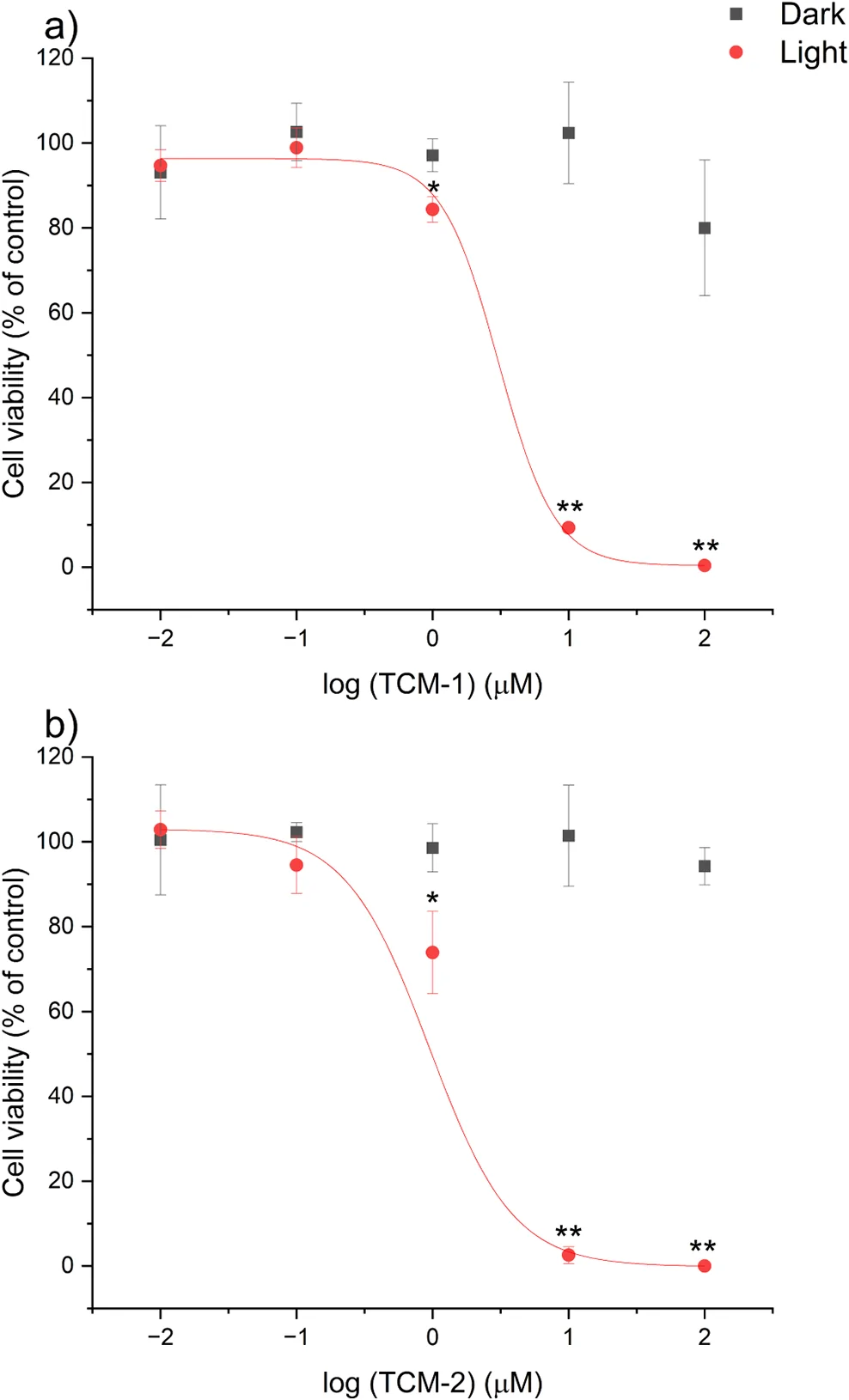
Cell viability values for a) **TCM-1** and b) **TCM-2** against normal fibroblast cells (1BR.3.G) kept in the dark (black squares) or exposed to the blue LED source (red dots). Data under light exposure were fitted to a dose-response curve (red line). Light dose was 12.9 J cm^–2^. Data represent the mean of three replicate biological samples and the SD. Statistical significance: *p < 0.05; **p < 0.01.

Phototoxicity results for **TCM-1** and **TCM-2** indicate a significant decrease in fibroblast viability at concentrations around 1–3 μM. These concentrations are lower than the 16 μM concentration of **TCM-2** required for substantial bacterial killing (Figure 4). This mismatch prevents the establishment of a therapeutic window, as the concentration needed to kill the bacteria would also entail significant toxicity in the surrounding tissue, even with a local application of light irradiation. Thus, the results of this preliminary test suggest that, in its current form, **TCM-2** may not yet be suitable for topical aPDT of skin infections, mainly because effective antibacterial activity requires relatively high concentrations. Nevertheless, these findings provide a valuable basis for further optimization. Future structural modifications will therefore focus on enhancing the antibacterial efficacy of these PSs at lower concentrations, with the ultimate goal of expanding their applicability to topical aPDT. Importantly, as highlighted in the Introduction, their promising photodynamic antibacterial activity supports their use as photosensitizing agents for the disinfection of surfaces in healthcare settings, while also establishing thiocoumarins as a promising molecular platform from which a new generation of PSs for aPDT could be developed. Interestingly, the observed phototoxicity toward eukaryotic cells may also encourage future studies to explore the potential of structurally optimized thiocoumarin derivatives in other photodynamic applications, including anticancer PDT.

## 3 Conclusions

In summary, this study establishes 2-thioxocoumarins as a previously unexplored molecular scaffold for antimicrobial photodynamic therapy. Thionation, combined with strategic bromination, profoundly modified the photophysical properties of the coumarin scaffold by extending absorption into the visible region, promoting ISC, and enabling efficient ROS generation via both Type I and Type II photochemical pathways. Among the synthesized derivatives, the monobrominated compound **TCM-2** displayed the most balanced combination of photophysical, photochemical, and biological properties, emerging as the lead PS for the photodynamic inactivation of both MSSA and MRSA. In contrast, the higher Type I ROS generation observed for the tribrominated (**TCM-3**–**TCM-4**) derivatives did not translate into superior antibacterial efficacy, demonstrating that photodynamic performance depends not only on ROS production but also on molecular features that govern solubility, aggregation, and interactions with bacterial cells. Although these compounds failed to show photodynamic activity against Gram-negative bacteria in this preliminary study, their outstanding performance against Gram-positive bacteria demonstrates the promise of this scaffold for antimicrobial photodynamic therapy. Ongoing research is therefore focused on the structural modification of coumarin and thiocoumarin derivatives to enhance their aPDT efficacy against Gram-negative pathogens.

This work provides fundamental structure-property-activity insights into sulfur-containing coumarins and demonstrates that rational modulation of the coumarin scaffold represents an effective strategy for developing new antimicrobial PSs. These findings establish 2-thioxocoumarins as promising antimicrobial PSs for surface disinfection in healthcare settings and, importantly, position this family of compounds as a valuable molecular scaffold for the rational design of next-generation antimicrobial PSs.

## 4 Experimental Section

### 4.1 Reagents and Materials

Selected reactions were carried out under nitrogen using standard Schlenk techniques. All reagents were purchased and used without further purification. Reaction progress was monitored by TLC using precoated silica gel plates (ALUGRAM SILG/UV254) and visualized under a UV254 lamp. Flash column chromatography was carried out on silica gel (230–400 mesh). Commercial-grade methanol, ethanol, acetone, tetrahydrofuran, dichloromethane, ethyl acetate, and hexane were used without further purification.

7-(Diethylamino)-2H-chromen-2-one (**CM-1**) and 3-bromo-7-(diethylamino)-2H-chromen-2-one (**CM-2**) were synthesized according to previously reported procedures, and their spectroscopic characterization was consistent with the reported data.^65^

### 4.2 Characterization Methods

Elemental analyses were obtained using a Thermo (Carlo Erba) Flash 2000 Elemental Analyzer, configured for wt% CHN. Infrared spectra were acquired using a JASCO FT-IR spectrophotometer, and wavenumbers are reported in cm^–1^. The ^1^H NMR (300 MHz) and ^13^C NMR (75 MHz) spectra were acquired using a Bruker DPX300 (300 MHz) spectrometer in DMSO-d_6_ or CDCl_3_ at room temperature. Chemical shifts are reported in ppm relative to the residual solvent signals for ^1^H and ^13^C NMR. All coupling constants are reported in hertz. UV–Vis absorption spectra were acquired using 8453 Agilent or JASCO V-770 UV–VIS spectrophotometers with 1 cm cuvettes, while the emission spectra were measured using an FS5 spectrofluorometer from Edinburgh Instruments. Samples were prepared in spectroscopic-grade solvents and adjusted to a response within the linear range. No fluorescent contaminants were detected on excitation in the wavelength region of experimental interest.

The *Φ*_f_ values of **CM-1**–**CM-4** were determined relative to quinine sulfate (*Φ*_f_ = 0.54 in 0.1 M H_2_SO_4_)^52^ as a standard and corrected for the refractive index of the solvent. Samples were prepared to achieve an absorbance around 0.1 at the excitation wavelength.

Dihydrorhodamine 123 (DHR-123) was used as a probe for ROS generation. For this purpose, mixtures of DHR-123 and the PSs were prepared in ACN (*c*_DHR‑123_ = 3.75 × 10^–5^ M, *c*_PS_ = 1.25 × 10^–5^ M). The formation of rhodamine 123, resulting from the oxidation of DHR-123 over time, was followed by measuring the increase in absorbance at 500 nm under LED irradiation at 405 nm (71 mW cm^–2^).

9,10-Dimethylanthracene (DMA) was employed as a chemical trap to evaluate the ^1^O_2_ photosensitizing properties of all PSs. For this purpose, mixtures of DMA and PSs were prepared in ACN (*c*_DMA_ = 7.5 × 10^–5^ M, *c*_PS_ = 2.5 × 10^–5^ M) and the changes in their absorption spectra were measured over time during irradiation at λ_exc_ = 450 nm (80 mW cm^–2^). The same experiment was carried out with perinaphthenone (PN) as a reference under the same solvent and irradiation conditions. From the apparent rate constants of ^1^O_2_-mediated DMA degradation derived from monoexponential fits of the time-dependent absorption data for both the PSs (*k*_PS_) and PN (*k*_PN_) (see Figure 3d), *Φ*_Δ_ values for each PS tested were determined according to eq 1:

1 $$\Phi_{\Delta ,\mathrm{PS}}=\Phi_{\Delta ,\mathrm{PN}}\frac{k_{\mathrm{PS}}\;A_{\mathrm{PN}}}{k_{\mathrm{PN}}\;A_{\mathrm{PS}}}$$

In this equation, *A*_PS_ and *A*_PN_ represent the absorbance values of the PS and PN solutions measured at the excitation wavelength (λ_exc_ = 450 nm), and *Φ*_Δ,PN_ is the literature-reported *Φ*_Δ_ value for PN in the selected solvent (*Φ*_Δ,PN_ = 1 in ACN).^53^ Each *Φ*_Δ_ value represents the mean of three independent replicates.

Irradiation experiments of bacterial cultures were performed using a Cameo STUDIO PAR 6 G2 LED spotlight, with the blue emission channel exclusively. The fluence rate (irradiance) of the LED source was determined with a calibrated optical power meter (LASERPOINT PC-PLUG A-2-D12-HPB-U, sensitivity 286.57 mV-7 W). The light dose (J/cm^2^) was calculated according to the following equation:

$$\mathrm{Dose}(J/{\mathrm{cm}}^{2})=\mathrm{Fluence}\;\mathrm{rate}(\mathrm{mW}/{\mathrm{cm}}^{2})\times \mathrm{Time}(s)\times 10^{-3}$$

Measurements were carried out with the LED source positioned above the bacteria-containing plate at a fixed distance to ensure homogeneous illumination.

### 4.3 Synthesis and Spectroscopic Characterization of Compounds

**Synthesis of 3,6,8-tribromo-7-(ethylamino)-2H-chromen-2-one (CM-3) and 3,6,8-tribromo-7-amino-2H-chromen-2-one (CM-4):** In a 25 mL round-bottom flask, 7-(diethylamino)-2H-chromen-2-one (**CM-1**) (600 mg, 2.8 mmol) was dissolved in acetic acid (10 mL). Bromine (Br_2_, 1.83 mL, 35.7 mmol) was added dropwise, and the reaction mixture was stirred at room temperature overnight. After completion of the reaction, the mixture was extracted with dichloromethane (3 × 10 mL) and washed with a 5% aqueous NaOH solution (15 mL). The organic phase was dried over anhydrous MgSO_4_, filtered, and concentrated under reduced pressure. Purification by column chromatography on silica gel using hexane/dichloromethane (6:4) as eluent afforded the major product **CM-3** (425 mg, 39% yield) and the minor product **CM-4** (232 mg, 20% yield), both obtained as yellow solids. **CM-3**: Elemental Analysis: Calculated for C_11_H_8_Br_3_NO_2_·0.1C_5_H_12_: C 31.89, H 2.14, N 3.33; experimental: C 31.73, H 2.11, N 3.30. IR (ATR, cm^–1^): 2981 (w), 2967 (w), 1704 (s), 1600 (s), 1236 (m), 1124 (m). ^1^H NMR (300 MHz, DMSO-d_6_, δ, ppm): 8.40 (s, 1H, H-4), 7.92 (s, 1H, H-5), 5.31 (t, J = 6.1 Hz, 1H, N–H), 3.53 (qt, J = 7.1 Hz, 2H, H-9), 1.14 (t, J = 7.1 Hz, 3H, H-10). ^13^C NMR (75 MHz, DMSO-d_6_, δ, ppm): 157.1 (C-2), 151.4 (C-8a), 148.5 (C-7), 144.8 (C-4), 131.7 (C-5), 114.6 (C-4a), 109.8 (C-6), 107.9 (C-3), 100.8 (C-8), 42.8 (C-9), 17.0 (C-10). **CM-4:** Elemental Analysis: Calculated for C_9_H_4_Br_3_NO_2_·0.2C_5_H_12_: C 29.13, H 1.56, N 3.40; experimental: C 29.29, H 1.56, N 3.25. IR (ATR, cm^–1^): 2967 (w), 2891 (w), 1702 (s), 1614 (s), 1243 (m), 1124 (m). ^1^H NMR (300 MHz, DMSO-d_6_, δ, ppm): 8.36 (s, 1H, H-4), 7.85 (s, 1H, H-5), 6.42 (s, 2H, N–H). ^13^C NMR (75 MHz, DMSO-d_6_, δ, ppm): 157.2 (C-2), 151.6 (C-8a), 147.5 (C-7), 145.4 (C-4), 130.9 (C-5), 111.8 (C-4a), 105.4 (C-3), 104.3 (C-6), 94.0 (C-8).

**Synthesis of 7-(diethylamino)-2H-chromen-2-thione (TCM-1):** In a 25 mL round-bottom flask, under a nitrogen atmosphere, 7-(diethylamino)-2H-chromen-2-one (**CM-1**) (100 mg, 0.46 mmol) and Lawesson’s reagent (186 mg, 0.46 mmol) were dissolved in dry toluene (3 mL). The reaction mixture was heated to reflux overnight. After completion of the reaction, the solvent was evaporated under reduced pressure, and the crude product was purified by column chromatography on silica gel using petroleum ether/dichloromethane (2:1) as the eluent, affording **TCM-1** (46 mg, 43% yield) as a red solid. Elemental Analysis: Calculated for C_13_H_15_NOS: C 66.92, H 6.48, N 6.00; experimental: C 66.67, H 6.21, N 5.90. IR (ATR, cm^–1^): 2968 (w), 2888 (w), 1614 (s), 1580 (s), 1237 (m), 1070 (m). ^1^H NMR (300 MHz, CDCl_3_, δ, ppm): 7.29 (d, J = 9.0 Hz, 1H, H-4), 7.28 (d, J = 8.8 Hz, 1H, H-5), 6.95 (d, J = 9.0 Hz, 1H, H-3), 6.70–6.64 (m, 2H, H-6, H-8), 3.46 (q, J = 7.1 Hz, 4H, H-9), 1.25 (t, J = 7.1 Hz, 6H, H-10). ^13^C NMR (75 MHz, CDCl_3_, δ, ppm): 197.6 (C-2), 159.4 (C-8a), 151.1 (C-7), 136.1 (C-4), 128.8 (C-5), 123.0 (C-3), 110.6 (C-4a), 110.3 (C-6), 97.0 (C-8), 44.9 (C-9, C-9́), 12.3 (C-10, C-10́).

**Synthesis of 3-bromo-7-(diethylamino)-2H-chromen-2-thione (TCM-2): TCM-2** was prepared according to the procedure described for **TCM-1**, starting from **CM-2** (300 mg, 1.0 mmol), Lawesson’s reagent (409 mg, 1.0 mmol), and dry toluene (6 mL). The crude product was purified by column chromatography on silica gel using hexane/dichloromethane (6:4) as the eluent, affording **TCM-2** (165 mg, 52% yield) as a red solid. Elemental Analysis: Calculated for C_13_H_14_BrNOS·0.2CH_2_Cl_2_: C 48.16, H 4.41, N 4.25; experimental: C 48.37, H 4.25, N 4.24. IR (ATR, cm^–1^): 2965 (w), 2896 (w), 1620 (s), 1567 (s), 1238 (m), 1078 (s). ^1^H NMR (300 MHz, CDCl_3_, δ, ppm): 7.84 (s, 1H, H-4), 7.25 (d, J = 8.7 Hz, 1H, H-5), 6.65 (dd, J_1_ = 8.7 Hz, J_2_ = 2.5 Hz, 1H, H-6), 6.62 (d, J = 2.5 Hz, 1H, H-8), 3.46 (q, J = 7.1 Hz, 4H, H-9), 1.26 (t, J = 7.1 Hz, 6H, H-10). ^13^C NMR (75 MHz, CDCl_3_, δ, ppm): 192.1 (C-2), 159.4 (C-8a), 151.5 (C-7), 138.3 (C-4), 128.3 (C-5), 116.8 (C-6), 111.0 (C-4a), 110.9 (C-3), 96.6 (C-8), 45.1 (C-9, C-9́), 12.4 (C-10, C-10́).

**Synthesis of 3,6,8-tribromo-7-(ethylamino)-2H-chromen-2-thione (TCM-3): TCM-3** was prepared according to the procedure described for **TCM-1**, starting from **CM-3** (200 mg, 0.47 mmol), Lawesson’s reagent (190 mg, 0.47 mmol), and dry toluene (3 mL). The crude product was purified by column chromatography on silica gel using hexane/dichloromethane (6:4) as the eluent, affording **TCM-3** (83 mg, 40% yield) as a red solid. Elemental Analysis: Calculated for C_11_H_8_Br_3_NOS·0.1C_5_H_12_: C 30.75, H 2.06, N 3.12; experimental: C 30.76, H 1.82, N 3.12. IR (ATR, cm^–1^): 3357 (w), 2970 (m), 2898 (m), 1616 (s), 1597 (s), 1236 (m), 1079 (m). ^1^H NMR (300 MHz, DMSO-d_6_, δ, ppm): 8.44 (s, 1H, H-4), 8.02 (s, 1H, H-5), 5.67 (t, J = 6.3 Hz, 1H, N–H), 3.64 (qt, J = 7.1 Hz, 2H, H-9), 1.18 (t, J = 7.1 Hz, 3H, H-10). ^13^C NMR (75 MHz, DMSO-d_6_, δ, ppm): 191.4 (C-2), 154.3 (C-8a), 148.6 (C-7), 137.8 (C-4), 131.5 (C-5), 120.5 (C-4a), 115.3 (C-6), 110.5 (C-3), 98.2 (C-8), 42.2 (C-9), 16.6 (C-10).

**Synthesis of 3,6,8-tribromo-7-amino-2H-chromen-2-thione (TCM-4): TCM-4** was prepared according to the procedure described for **TCM-1**, starting from **CM-4** (350 mg, 0.88 mmol), Lawesson’s reagent (355 mg, 0.88 mmol), and dry toluene (6 mL). The crude product was purified by column chromatography on silica gel using hexane/dichloromethane (6:4) as the eluent, affording **TCM-4** (185 mg, 51% yield) as a red solid. Elemental Analysis: Calculated for C_9_H_4_Br_3_NOS·0.05C_5_H_12_: C 26.61, H 1.10, N 3.35; experimental: C 26.79, H 1.03, N 3.42. IR (ATR, cm^–1^): 3449 (s), 3313 (m), 2978 (m), 2903 (m), 1608 (m), 1560 (m), 1228 (w), 1075 (w). ^1^H NMR (300 MHz, DMSO-d_6_, δ, ppm): 8.37 (s, 1H, H-4), 7.93 (s, 1H, H-5), 6.70 (s, 2H, N–H). ^13^C NMR (75 MHz, DMSO-d_6_, δ, ppm): 191.6 (C-2), 154.8 (C-8a), 148.4 (C-7), 138.9 (C-4), 131.2 (C-5), 119.2 (C-4a), 113.5 (C-6), 106.3 (C-3), 93.1 (C-8).

### 4.4 X-ray Crystallographic Analysis

Crystals of **TCM-2**, **TCM-3**, and **TCM-4** were obtained from the slow evaporation of chloroform solutions. Single-crystal X-ray diffraction data sets for **TCM-2**, **TCM-3,** and **TCM-4** were acquired at 100 K on a Bruker D8 Venture diffractometer with a frame separation of 0.3°. Data integration was carried out with SAINT V6.45A and SORTAV^66^ in the diffractometer package.^67^ The crystal and collection data together with the structural refinement parameters are summarized in Tables S1–S15 of the Supporting Information (SI). The structures were solved by direct methods with SHELXT-2014 and Fourier difference methods and subsequently refined against F^2^ by least-squares methods with SHELXL-2014/7 within the WinGX program environment.^68,69^ Anisotropic displacement parameters were applied to non-H atoms, whereas H atoms were placed at calculated positions and refined using a riding model relative to their parent atoms. Atomic coordinates, bond distances, and angles are provided in the corresponding tables in the SI.

Crystallographic data for these structures have been deposited in the Cambridge Crystallographic Data Centre under CCDC deposition numbers 2570423, 2570424, and 2570425. Copies of this information may be obtained free of charge from The Director, CCDC, 12 Union Road, Cambridge CB2 1EZ, UK. Fax: +44 1223 336 033. E-mail: data_request@ccdc.cam.ac.uk. Web page: http://www.ccdc.cam.ac.uk.

### 4.5 Biological Evaluation

#### 4.5.1 Bacterial Strains and Culture Conditions

Strains used in this study were: methicillin-sensitive*Staphylococcus aureus* (MSSA, ATCC 35556), gentamicin- and methicillin-resistant *Staphylococcus aureus*­(MRSA, ATCC 33592), *Escherichia coli* (ATCC 25922), and *Pseudomonas aeruginosa* (ATCC 10145). All strains were cultured on Luria-Bertani (LB) agar at 37 °C.

#### 4.5.2 Antimicrobial Susceptibility Testing

All compounds were initially solubilized in sterile DMSO at a concentration of 51.2 mM and kept at 4 °C in the dark until further use. Prior to antimicrobial susceptibility testing, all compounds were thawed and further diluted with cation-adjusted Mueller-Hinton broth (cMHB) to achieve a final DMSO concentration of 1%.

MIC values were determined using a broth microdilution assay against the four previously mentioned strains. Briefly, overnight cultures of each strain were diluted in cMHB to a final concentration of 1 × 10^6^ CFU/mL. 50 μL of the bacterial suspensions were added to a 96-well plate containing 50 μL of two-fold serial dilutions of each compound, resulting in a final inoculum concentration of 5 × 10^5^ CFU/mL and a maximum DMSO concentration of ≤0.5% at the highest tested compound concentration. Growth (cMHB + 0.5% DMSO) and sterility (no bacteria) controls were also included. All plates were prepared in duplicate. After 5 min of incubation in the dark, one of the plates was exposed to blue LED irradiation for 10 min, corresponding to a light dose of 25.7 J cm^–2^. Plates were subsequently incubated in the dark at 37 °C for 24 h. MIC end points were assessed as inhibition of visible growth. Each experiment included three technical replicates and was independently repeated three times.

#### 4.5.3 Dose-Response Curves

Dose-response assays were conducted to determine the effect of compound concentration on bacterial survival. The assays were carried out against MSSA and MRSA using **TCM-1** and **TCM-2**. Compounds and bacterial inocula were prepared as described in the previous section, but the initial bacterial concentration was adjusted to ∼2 × 10^8^ CFU/mL.

50 μL of the bacterial inocula were added to a 96-well plate containing 50 μL of two-fold serial dilutions of each compound in cMHB, achieving a final inoculum concentration of ∼1 × 10^8^ CFU/mL. For **TCM-1**, the tested concentrations were 128 μM, 64 μM, 32 μM, and 16 μM. For **TCM-2**, the tested concentrations were 16 μM, 8 μM, 4 μM, and 2 μM. For both compounds, a control condition with no compound was also included. All plates were prepared in duplicate. After 5 min of incubation in the dark, one of the plates was illuminated with a blue LED source for 10 min, delivering a light dose of 25.7 J cm^–2^.

All conditions were subsequently serially diluted down to 10^–4^ in phosphate-buffered saline (PBS), and 10 μL of each sample was plated on LB agar, using the track dilution method to obtain colony counts.^70^ Statistical significance was assessed using an unpaired t-test (OriginPro 2023b; version 10.0.5.153).

#### 4.5.4 Cell Lines and Culture Conditions

The cell line used in this study was 1BR.3.G (ECACC, UK). Cells were routinely cultured in Dulbecco’s Modified Eagle Medium (DMEM) + 10% fetal bovine serum (FBS) at 37 °C and 5% CO_2_. Cells were passaged twice per week.

#### 4.5.5 Cell Viability Study

1BR.3.G cells were seeded on a 96-well plate in duplicate at a density of 31250 cells/cm^2^ and incubated at 37 °C for 24 h. Afterward, several dilutions of **TCM-1** and **TCM-2** in DMEM were dispensed to the cells and incubated for 1 h at 37 °C. Then, one plate was exposed to the blue LED source for 5 min, achieving a light dose of 12.9 J cm^–2^. The other plate was kept in the dark. Each plate contained its own positive and negative controls.

Cells were incubated for up to 24 h after TCM exposure. Then, the TCM-containing medium was removed, and the cells were washed with PBS. Subsequently, a 50 μM solution of resazurin (Thermo Fisher Scientific, USA) in DMEM was added to each well, and cells were incubated at 37 °C for 4 h. Medium was then transferred to a black plate, and fluorescence was measured at λ_ex_ = 545 nm and λ_em_ = 590 nm using a SpectraMax iD3 Multi-Mode Microplate Reader (Molecular Devices, USA). Cell viability data were min-max normalized using untreated cells and cells treated with 20% DMSO as controls. In the plate illuminated under the blue LED, both controls were exposed to the same light dose as the cells treated with the compounds, to account for the possible effect of light exposure on cell viability. The IC_50_ values were determined by fitting a dose-response curve to data obtained from the light-treated cells, and statistical significance was assessed using an unpaired t-test (OriginPro 2023b; version 10.0.5.153).

## Supplementary Material



## Notes

^1^H and ^13^C NMR spectra; photophysical and photochemical characterization, including fluorescence spectroscopy and Type I and Type II ROS generation studies; X-ray crystallographic data for **TCM-2**–**TCM-4** (PDF)
