# Supplementary material for: Introducing 2‑Thioxocoumarins as a New Class of Photosensitizers for Antimicrobial Photodynamic Therapy
Source: ACS Omega. 2026 Oct 2;11(41):63755–67. doi: 10.1021/acsomega.6c07980 (PMC13688472; doi:10.1021/acsomega.6c07980)
Supplement: Supplementary file 1 [file ao6c07980_si_001.pdf]

Introducing 2-Thioxocoumarins as a New Class of Photosensitizers for Antimicrobial Photodynamic Therapy

Javier Ordóñez-Hernández [a*], Abel Jaume-Masferrer [a], Jordi Hernando [b], Marc Martínez-Miguel [a], Claire Brazzi [a], Elies Molins [a], Rosario Núñez [a*]

[a]*Institut de Ciència de Materials de Barcelona (ICMAB-CSIC), Carrer dels Til·lers, 08193 Cerdanyola del Vallès, Spain.*

[b]*Departament de Química. Universitat Autònoma de Barcelona, (UAB), 08193 Bellaterra (Cerdanyola del Vallès), Barcelona, Spain.*

To whom correspondence should be addressed. Email: jordonez@icmab.es and rosario@icmab.es

CONTENTS

1. Nuclear Magnetic Resonance Spectra

Figure S1. ^{1}H NMR spectrum of **CM-1** in $CDCl_3$ at 300 MHz 1

Figure S2. ^{13}C NMR spectrum of **CM-1** in $CDCl_3$ at 75 MHz 1

Figure S3. ^{1}H NMR spectrum of **CM-2** in $CDCl_3$ at 300 MHz 2

Figure S4. ^{13}C NMR spectrum of **CM-2** in $CDCl_3$ at 75 MHz 2

Figure S5. ^{1}H NMR spectrum of **CM-3** in DMSO-d_6 at 300 MHz 3

Figure S6. ^{13}C NMR spectrum of **CM-3** in DMSO-d_6 at 75 MHz 3

Figure S7. ^{1}H NMR spectrum of **CM-4** in DMSO-d_6 at 300 MHz 4

Figure S8. ^{13}C NMR spectrum of **CM-4** in DMSO-d_6 at 75 MHz 4

Figure S9. ^{1}H NMR spectrum of **TCM-1** in $CDCl_3$ at 300 MHz 5

Figure S10. ^{13}C NMR spectrum of **TCM-1** in $CDCl_3$ at 75 MHz 5

Figure S11. ^{1}H NMR spectrum of **TCM-2** in $CDCl_3$ at 300 MHz 6

Figure S12. ^{13}C NMR spectrum of **TCM-2** in $CDCl_3$ at 75 MHz 6

Figure S13. ^{1}H NMR spectrum of **TCM-3** in DMSO-d_6 at 300 MHz 7

Figure S14. ^{13}C NMR spectrum of **TCM-3** in DMSO-d_6 at 75 MHz 7

Figure S15. ^{1}H NMR spectrum of **TCM-4** in DMSO-d_6 at 300 MHz 8

Figure S16. ^{13}C NMR spectrum of **TCM-4** in DMSO-d_6 at 75 MHz 8

2. Photophysical and Photochemical Characterization

2.1 Fluorescence Spectroscopy

Figure S17. Fluorescence emission spectra of **CM-1** – **CM-4** 9

2.2 Evaluation of the Type I Mechanism

Figure S18. Variation of the absorption spectrum of a mixture of **TCM-1** + DHR-123 10

Figure S19. Variation of the absorption spectrum of a mixture of **TCM-2** + DHR-123 10

Figure S20. Variation of the absorption spectrum of a mixture of **TCM-3** + DHR-123 11

Figure S21. Variation of the absorption spectrum of a mixture of **TCM-4** + DHR-123 11

2.3. Evaluation of the Type II Mechanism

Figure S22. Variation of the absorption spectrum of a mixture of **TCM-1** + DMA 12

Figure S23. Variation of the absorption spectrum of a mixture of **TCM-2** + DMA 12

Figure S24. Variation of the absorption spectrum of a mixture of **TCM-3** + DMA 13

Figure S25. Variation of the absorption spectrum of a mixture of **TCM-4** + DMA 13

Figure S26. Variation of the absorption spectrum of a mixture of **PN** + DMA 14

3. X-ray Crystallographic Analysis

Table S1. Crystal data and structure refinement for **TCM-2** 15

Table S2. Atomic coordinates (× 10^4) and equivalent isotropic displacement parameters (Å^2 × 10^3) for **TCM-2**. 16

Table S3. Bond lengths [Å] and angles [°] for **TCM-2** 17

Table S4. Anisotropic displacement parameters (Å^2 × 10^3) for **TCM-2** 20

Table S5. Hydrogen bonds for **TCM-2** [Å and °] 21

Table S6. Crystal data and structure refinement for **TCM-3** 22

Table S7. Atomic coordinates (× 10^4) and equivalent isotropic displacement parameters (Å^2 × 10^3) for **TCM-3** 23

Table S8. Bond lengths [Å] and angles [°] for **TCM-3** 24

Table S9. Anisotropic displacement parameters (Å^2 × 10^3) for **TCM-3** 26

Table S10. Hydrogen bonds for **TCM-3** [Å and °] 27

Table S11. Crystal data and structure refinement for **TCM-4** 28

Table S12. Atomic coordinates (× 10^4) and equivalent isotropic displacement parameters (Å^2 × 10^3) for **TCM-4** 29

Table S13. Bond lengths [Å] and angles [°] for **TCM-4** 30

Table S14. Anisotropic displacement parameters (Å^2 × 10^3) for **TCM-4** 32

Table S15. Hydrogen bonds for **TCM-4** [Å and °] 33

1. Nuclear Magnetic Resonance Spectra

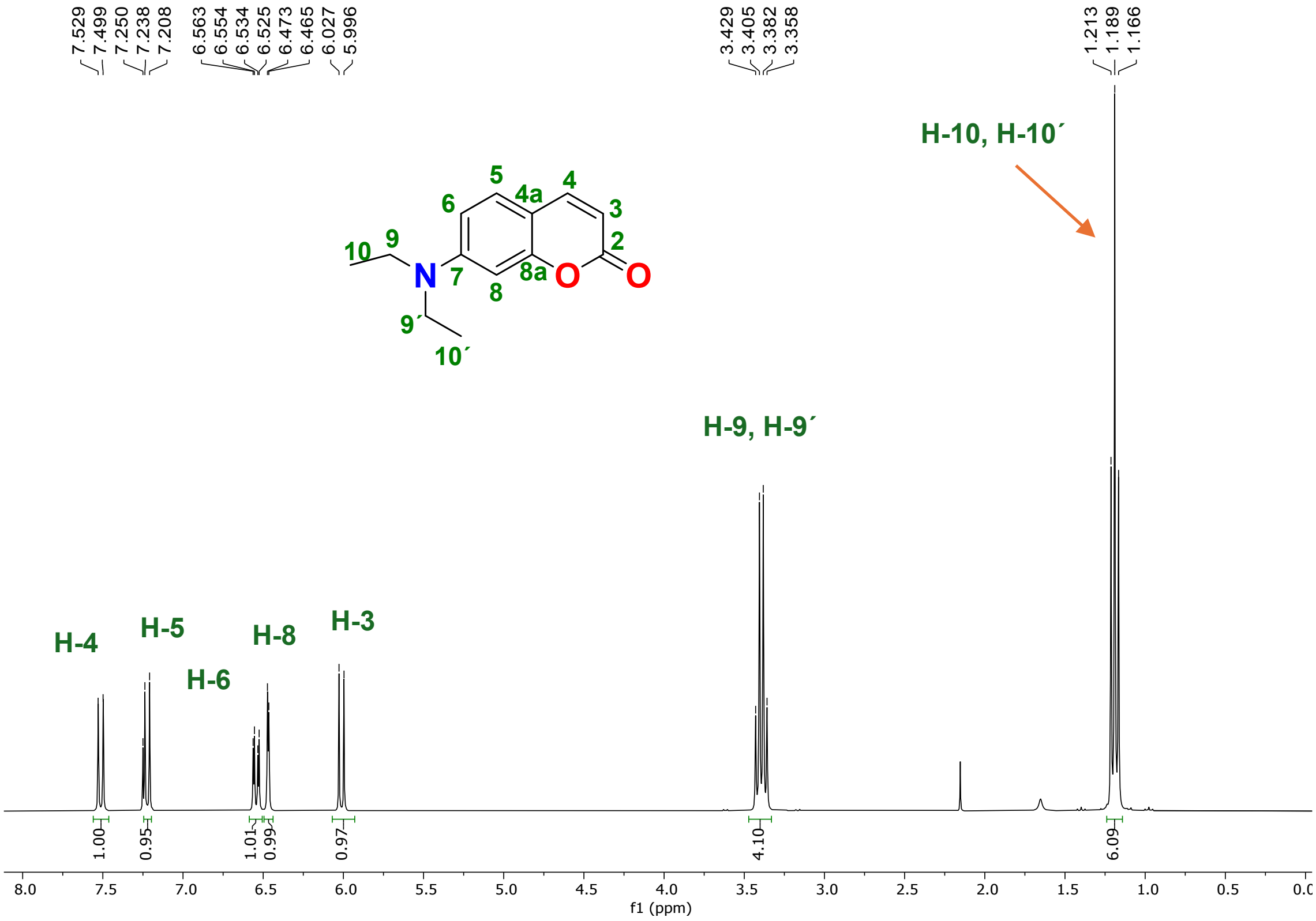

Figure S1. ^{1}H NMR spectrum of **CM-1** in $CDCl_3$ at 300 MHz.

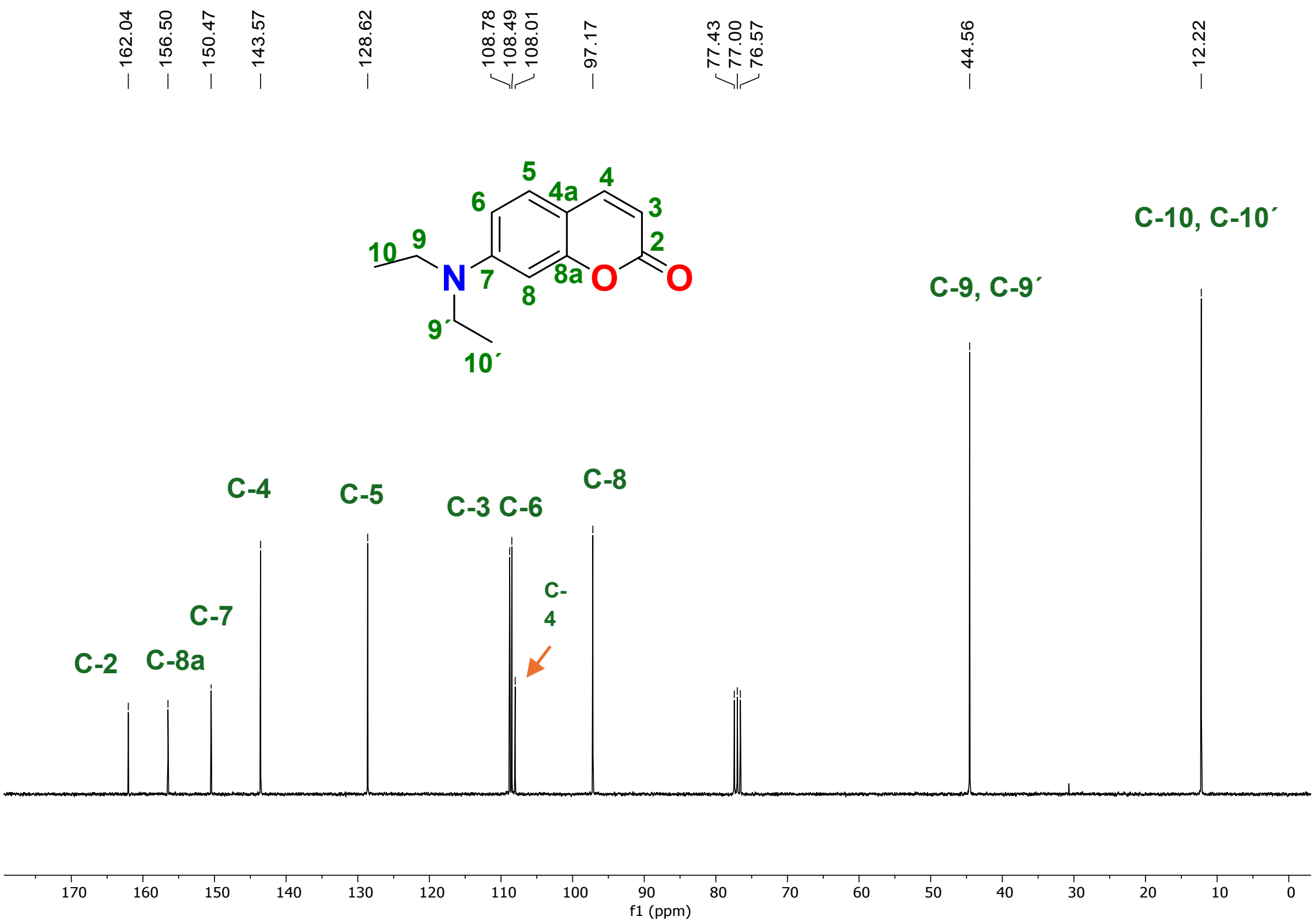

Figure S2. ^{13}C NMR spectrum of **CM-1** in $CDCl_3$ at 75 MHz.

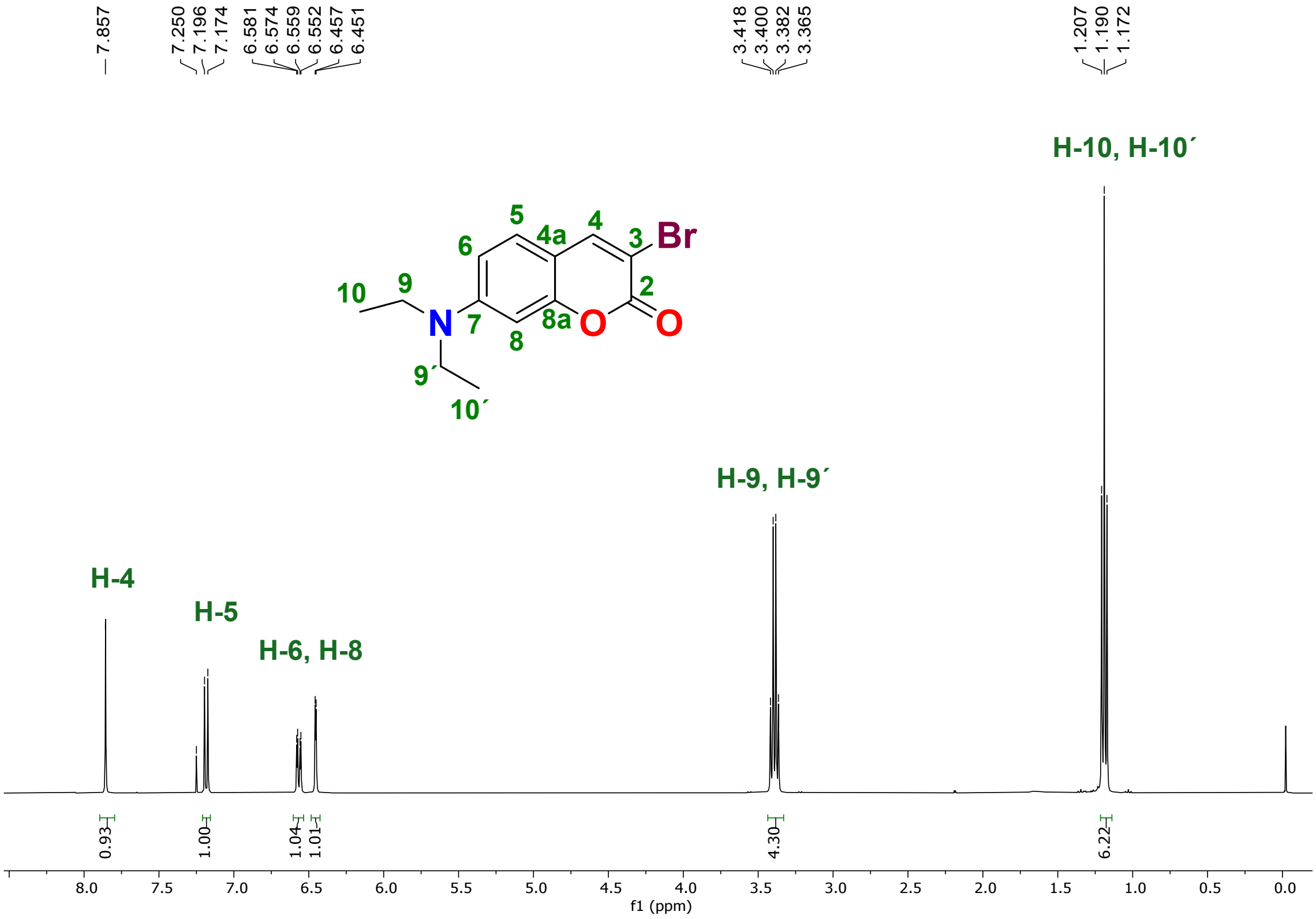

Figure S3. ^{1}H NMR spectrum of **CM-2** in $CDCl_3$ at 300 MHz.

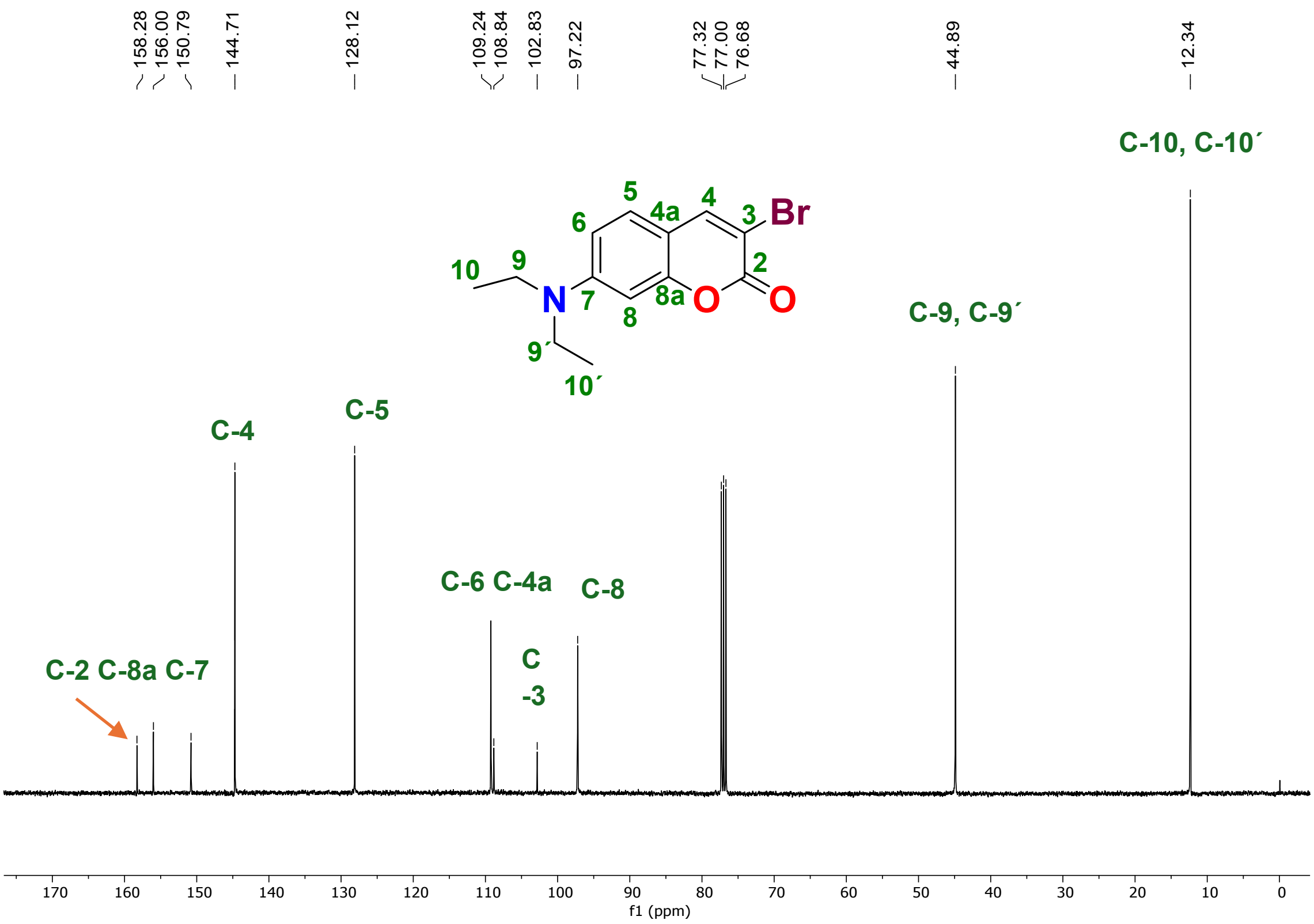

Figure S4. ^{13}C NMR spectrum of **CM-2** in $CDCl_3$ at 75 MHz.

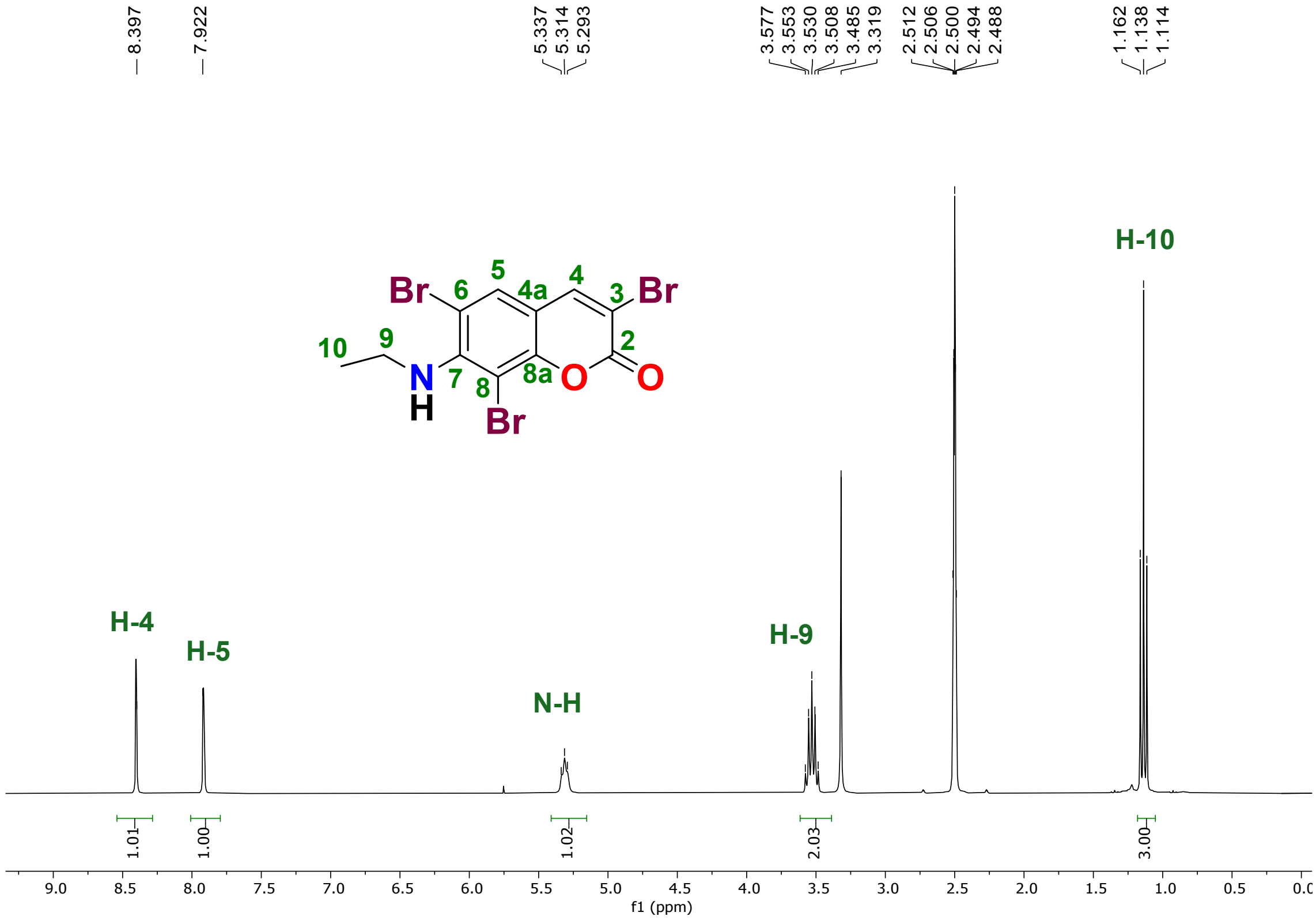

Figure S5. ^{1}H NMR spectrum of **CM-3** in DMSO-d_6 at 300 MHz.

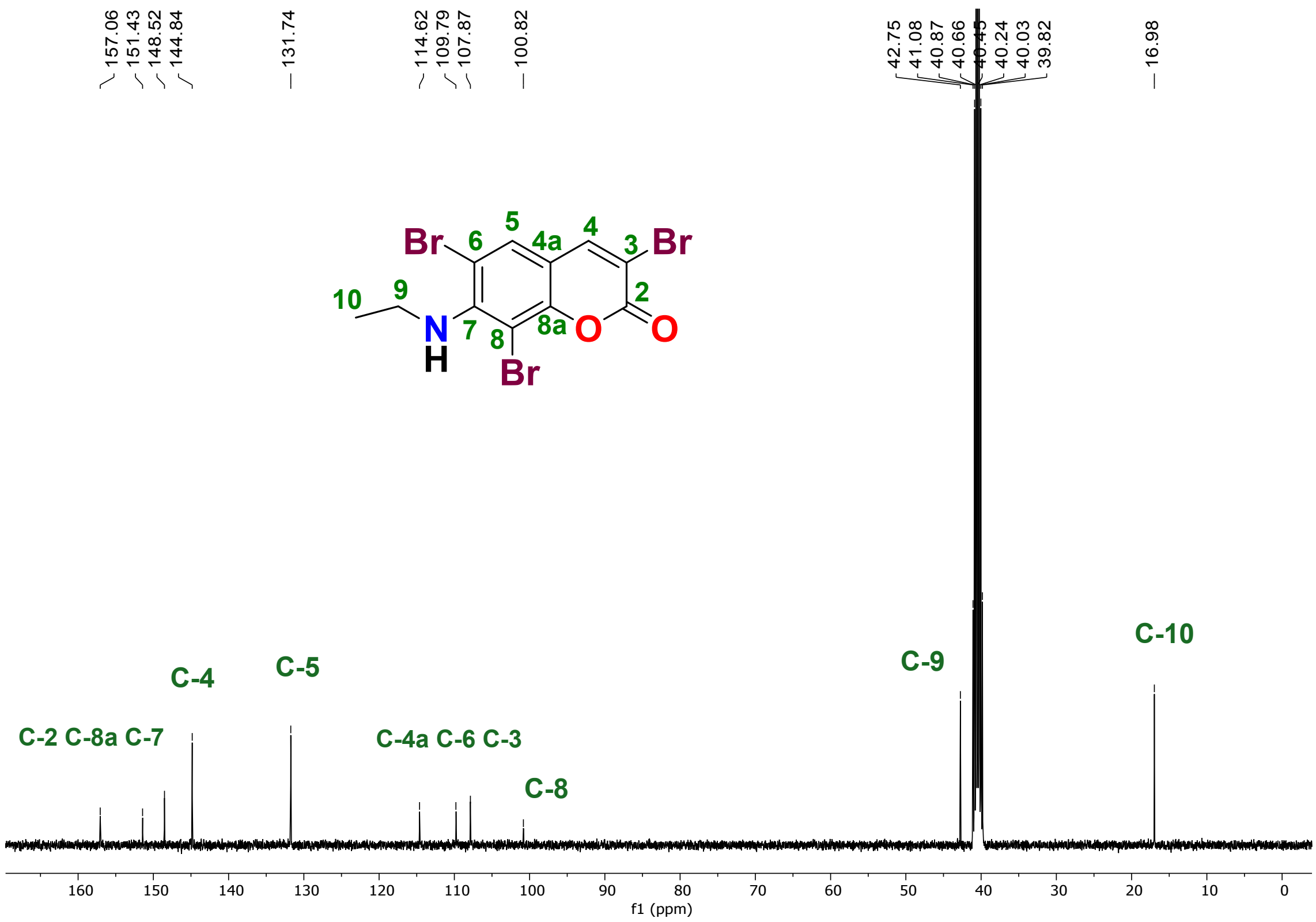

Figure S6. ^{13}C NMR spectrum of **CM-3** in DMSO-d_6 at 75 MHz.

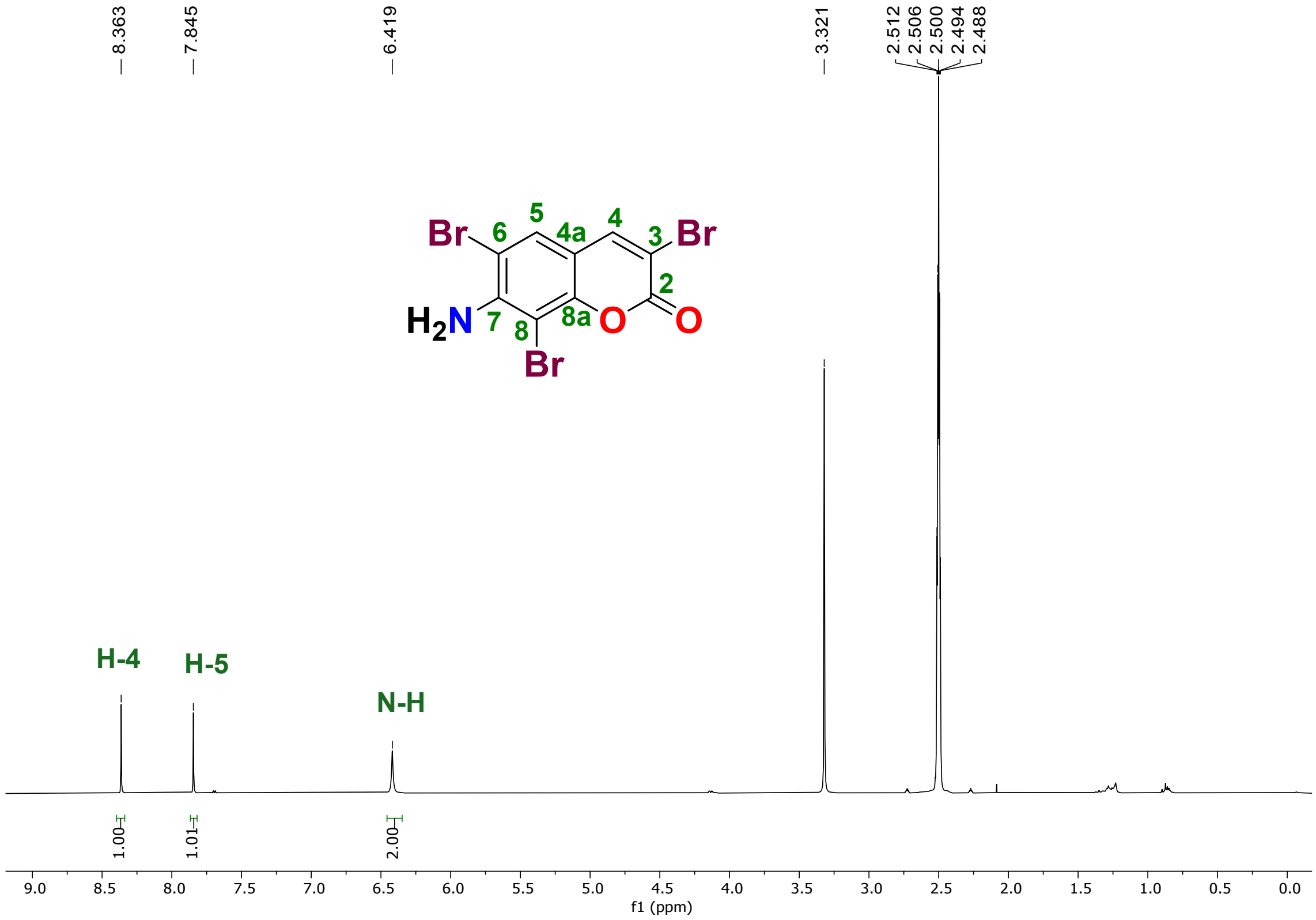

Figure S7. ^{1}H NMR spectrum of **CM-4** in DMSO-d$_6$ at 300 MHz.

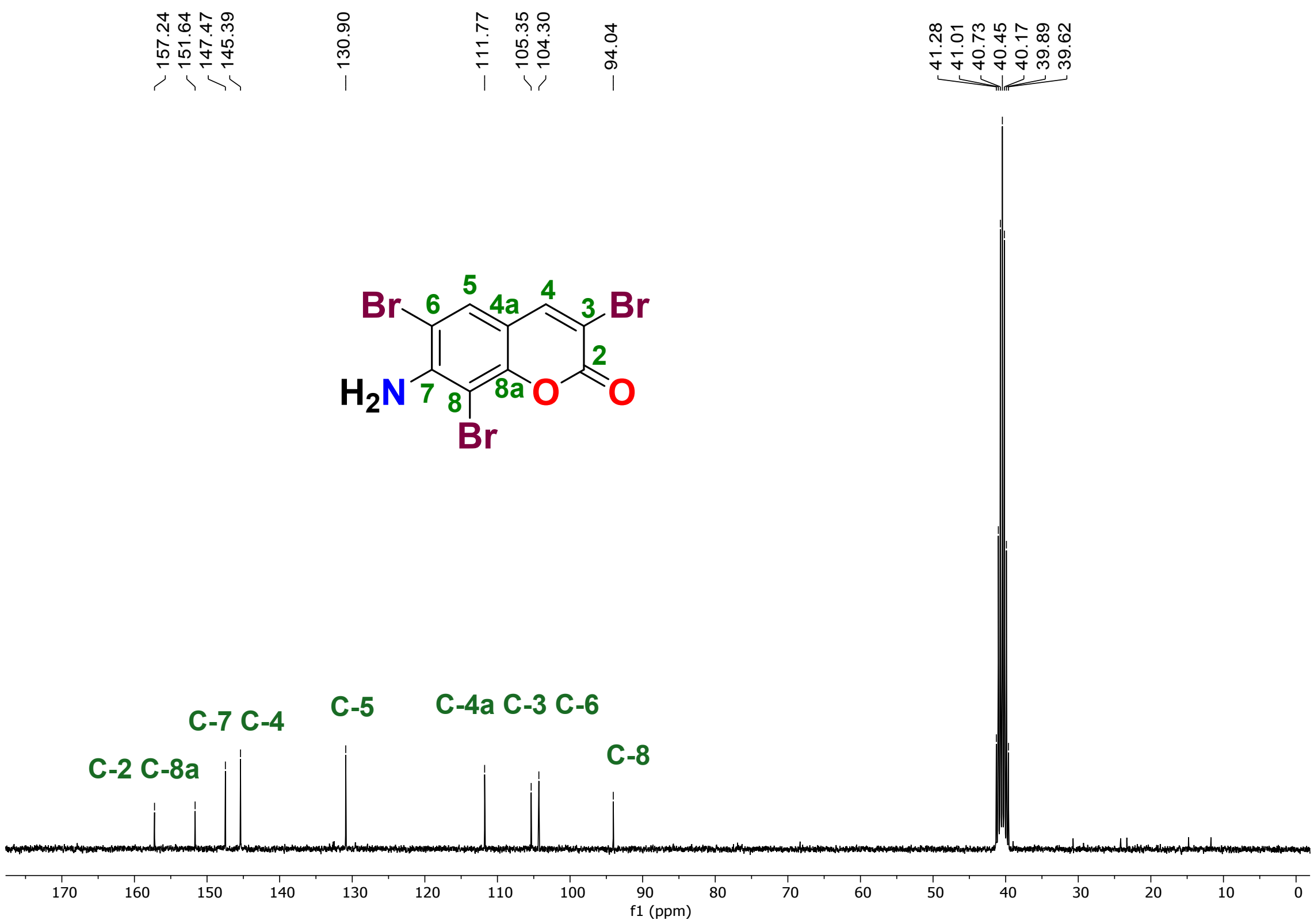

Figure S8. ^{13}C NMR spectrum of **CM-4** in DMSO-d$_6$ at 75 MHz.

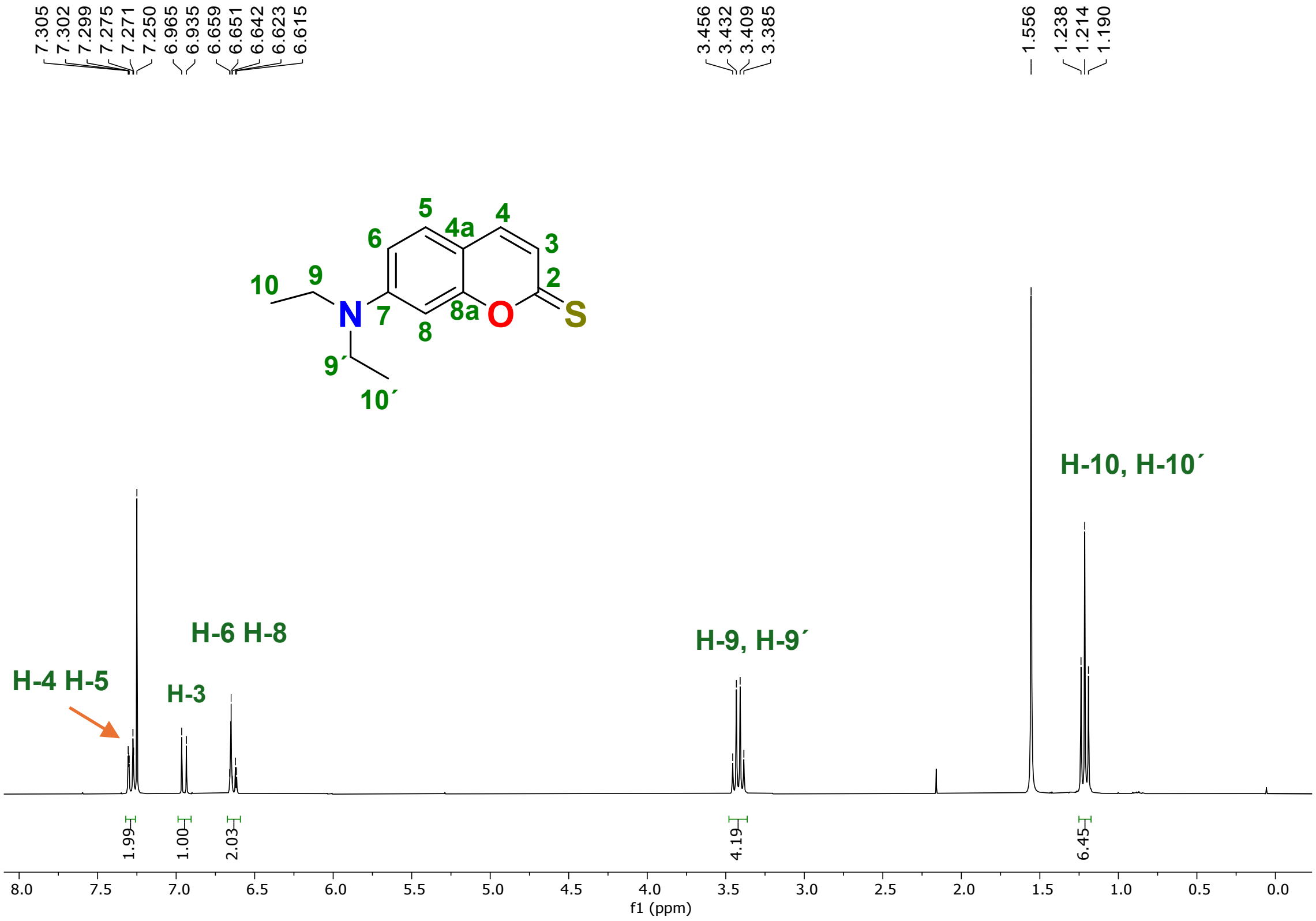

Figure S9. ^{1}H NMR spectrum of **TCM-1** in $CDCl_3$ at 300 MHz.

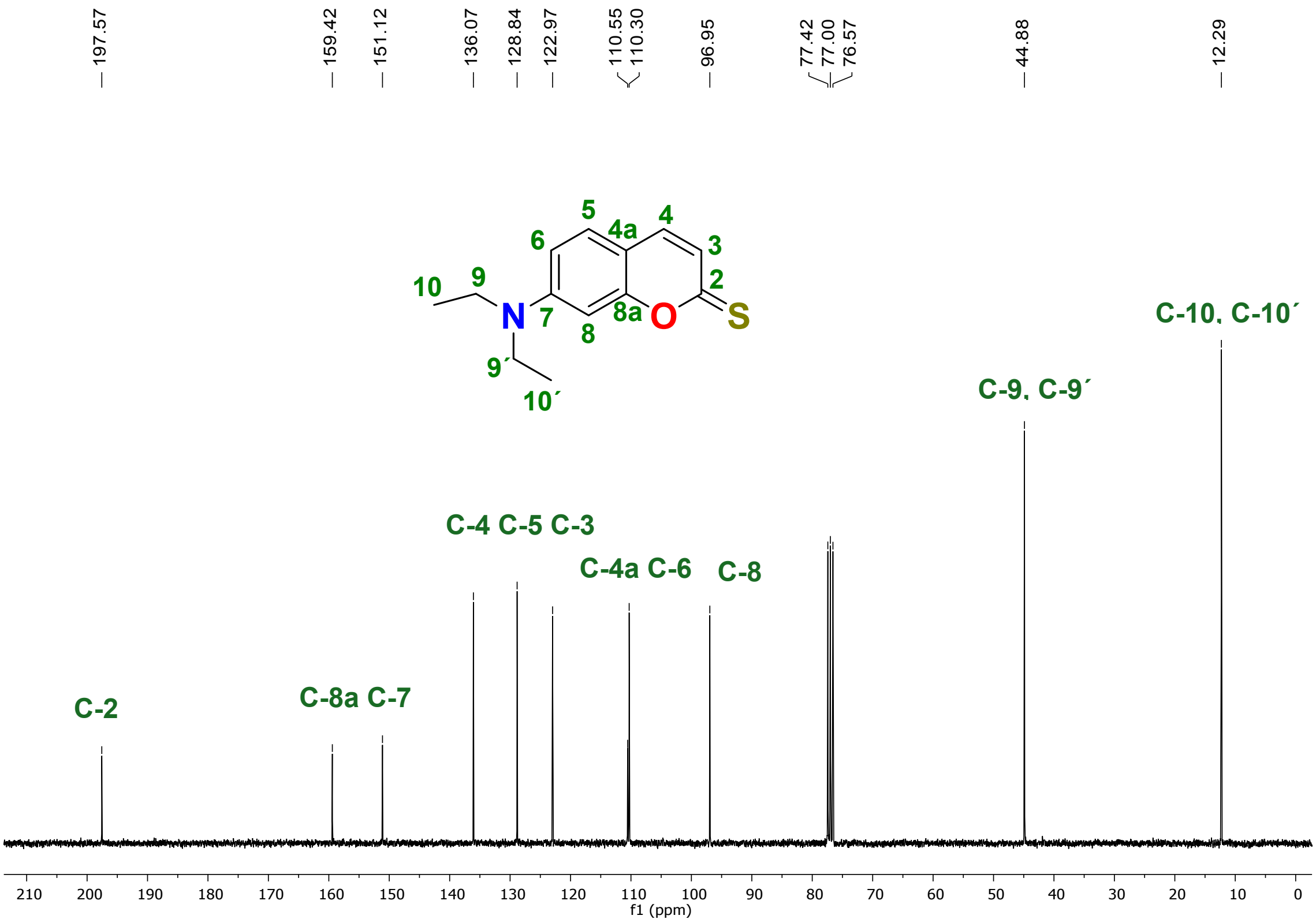

Figure S10. ^{13}C NMR spectrum of **TCM-1** in $CDCl_3$ at 75 MHz.

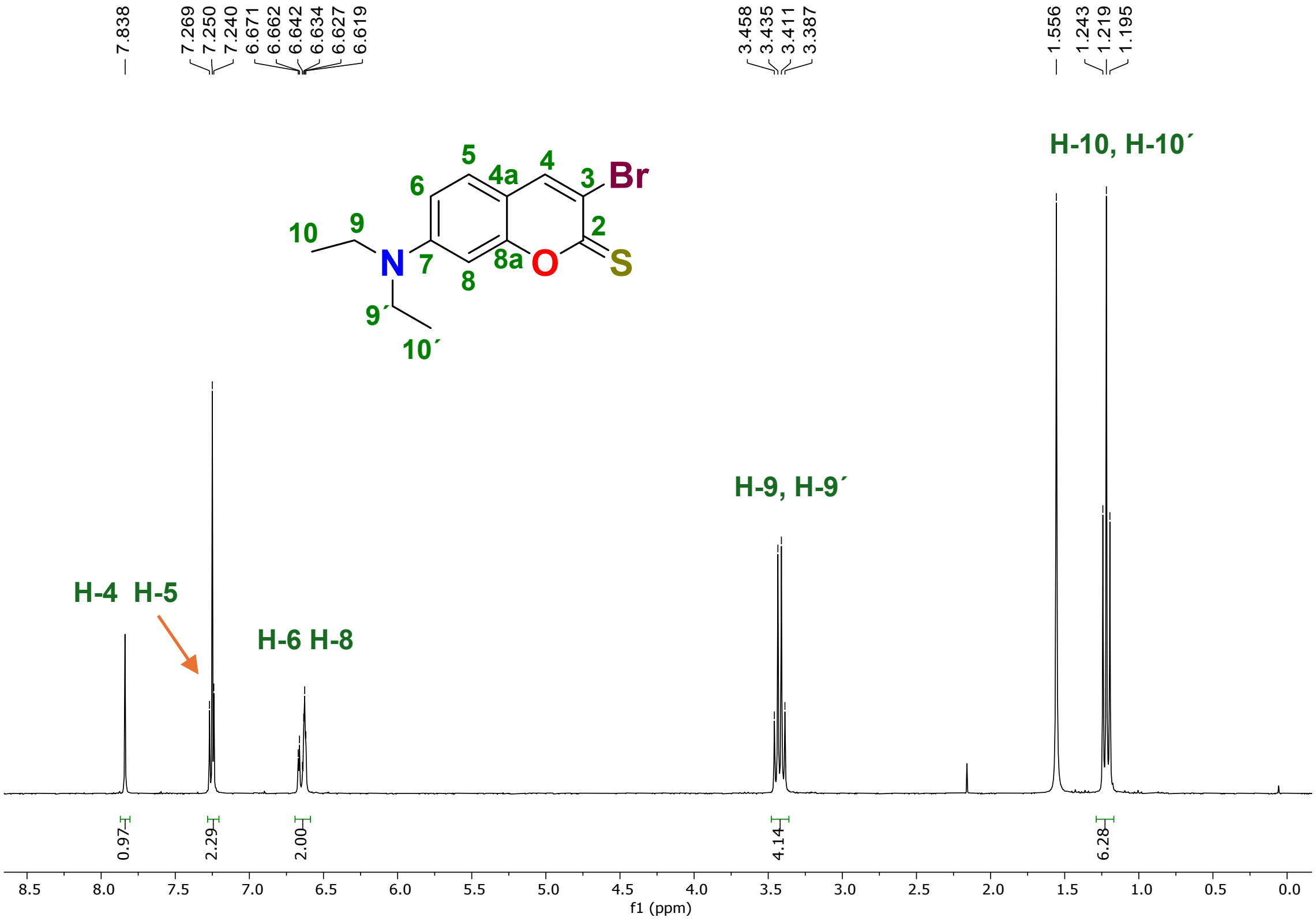

Figure S11. ^{1}H NMR spectrum of **TCM-2** in $CDCl_3$ at 300 MHz.

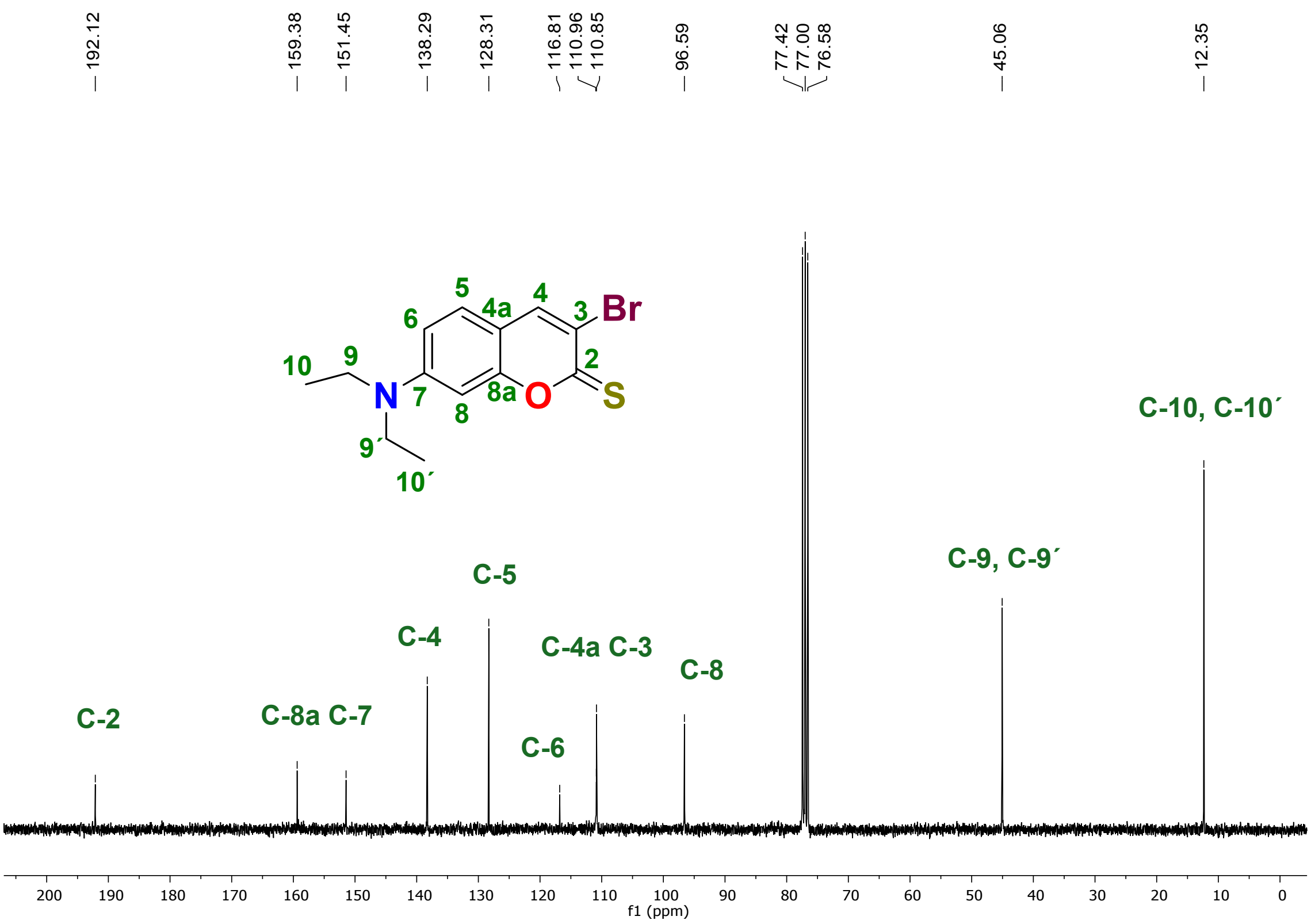

Figure S12. ^{13}C NMR spectrum of **TCM-2** in $CDCl_3$ at 75 MHz.

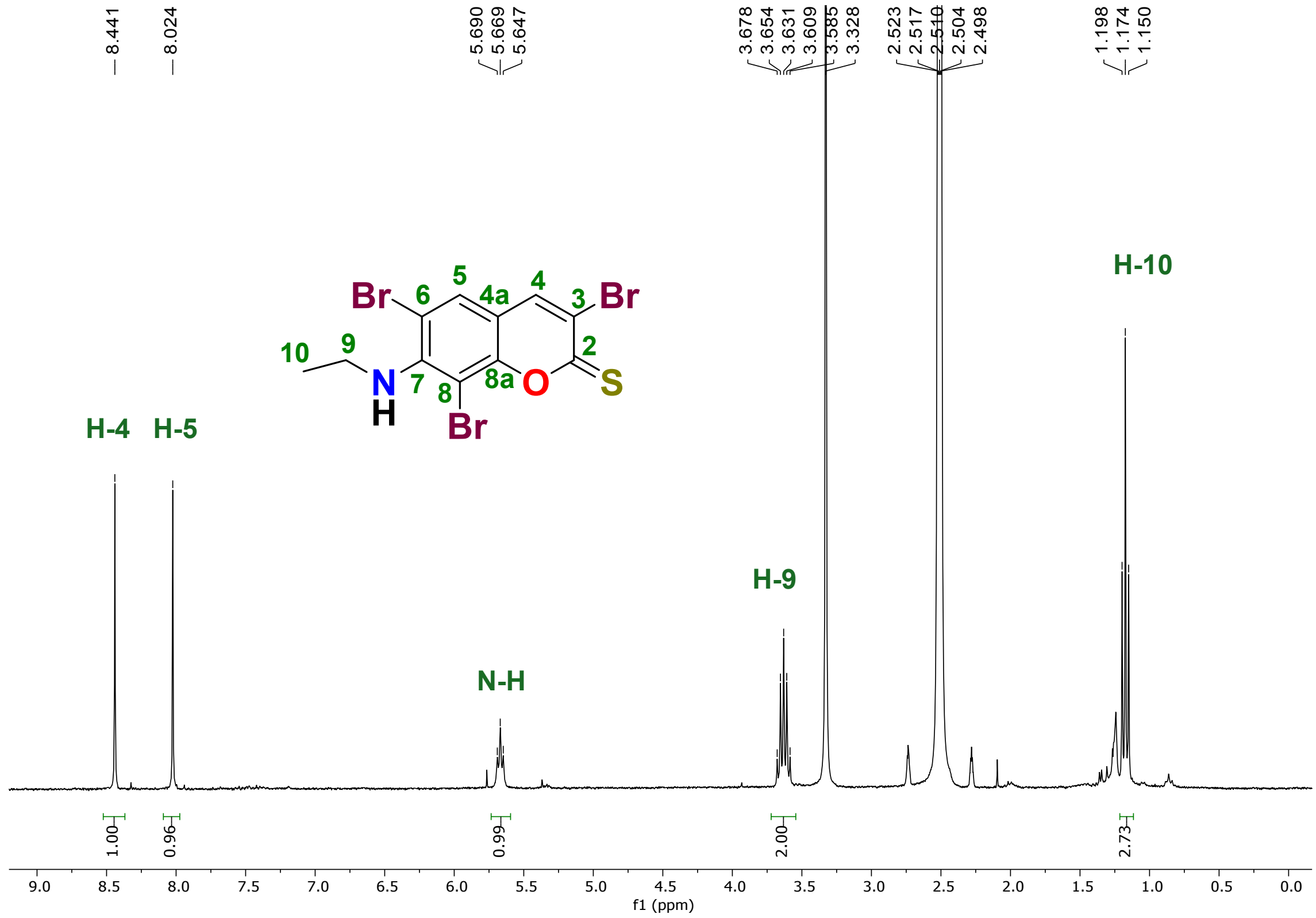

Figure S13. ^{1}H NMR spectrum of **TCM-3** in DMSO-d_6 at 300 MHz.

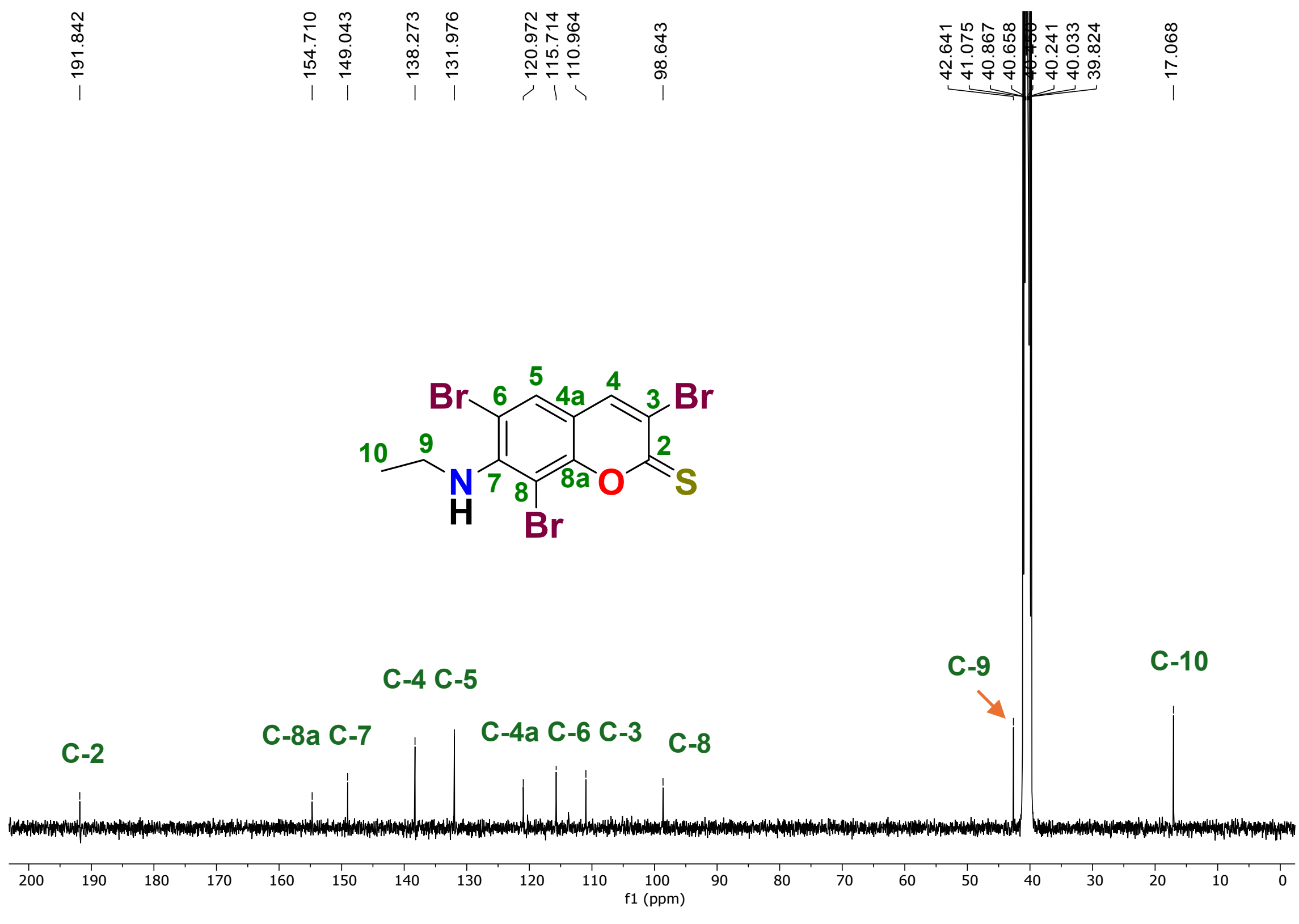

Figure S14. ^{13}C NMR spectrum of **TCM-3** in DMSO-d_6 at 75 MHz.

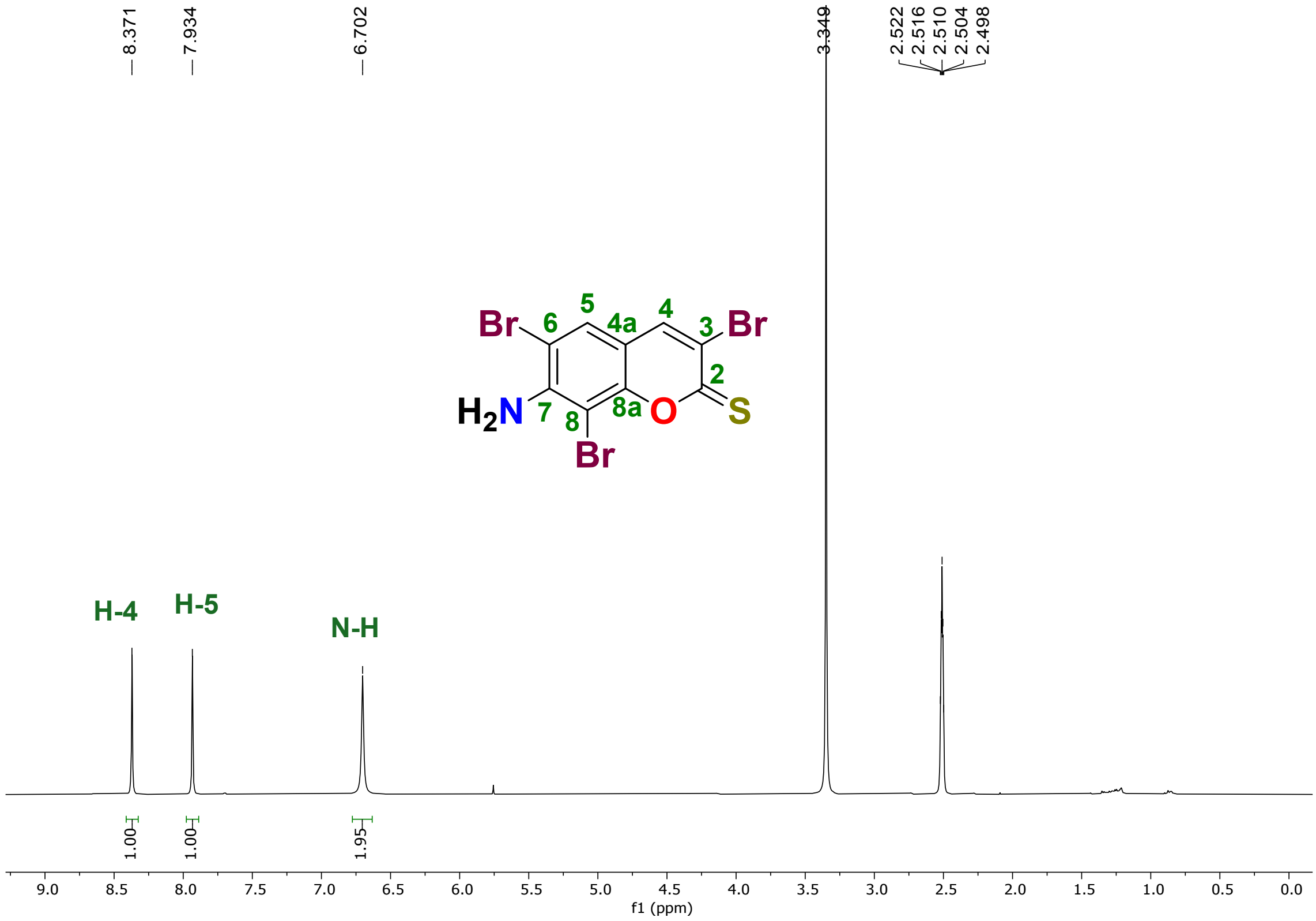

Figure S15. ^{1}H NMR spectrum of **TCM-4** in DMSO-d_6 at 300 MHz.

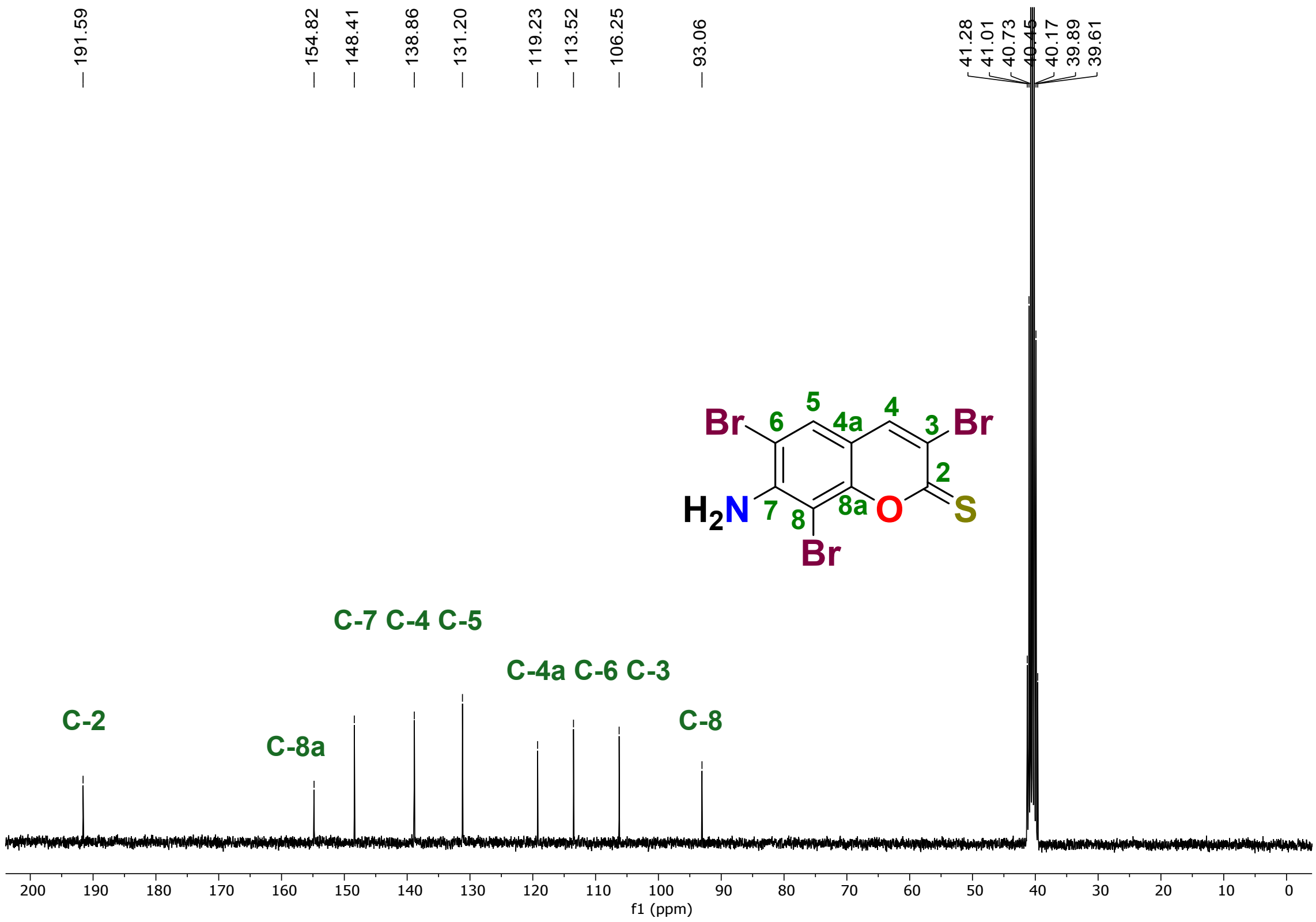

Figure S16. ^{13}C NMR spectrum of **TCM-4** in DMSO-d_6 at 75 MHz.

2. Photophysical and Photochemical Characterization

2.1 Fluorescence Spectroscopy

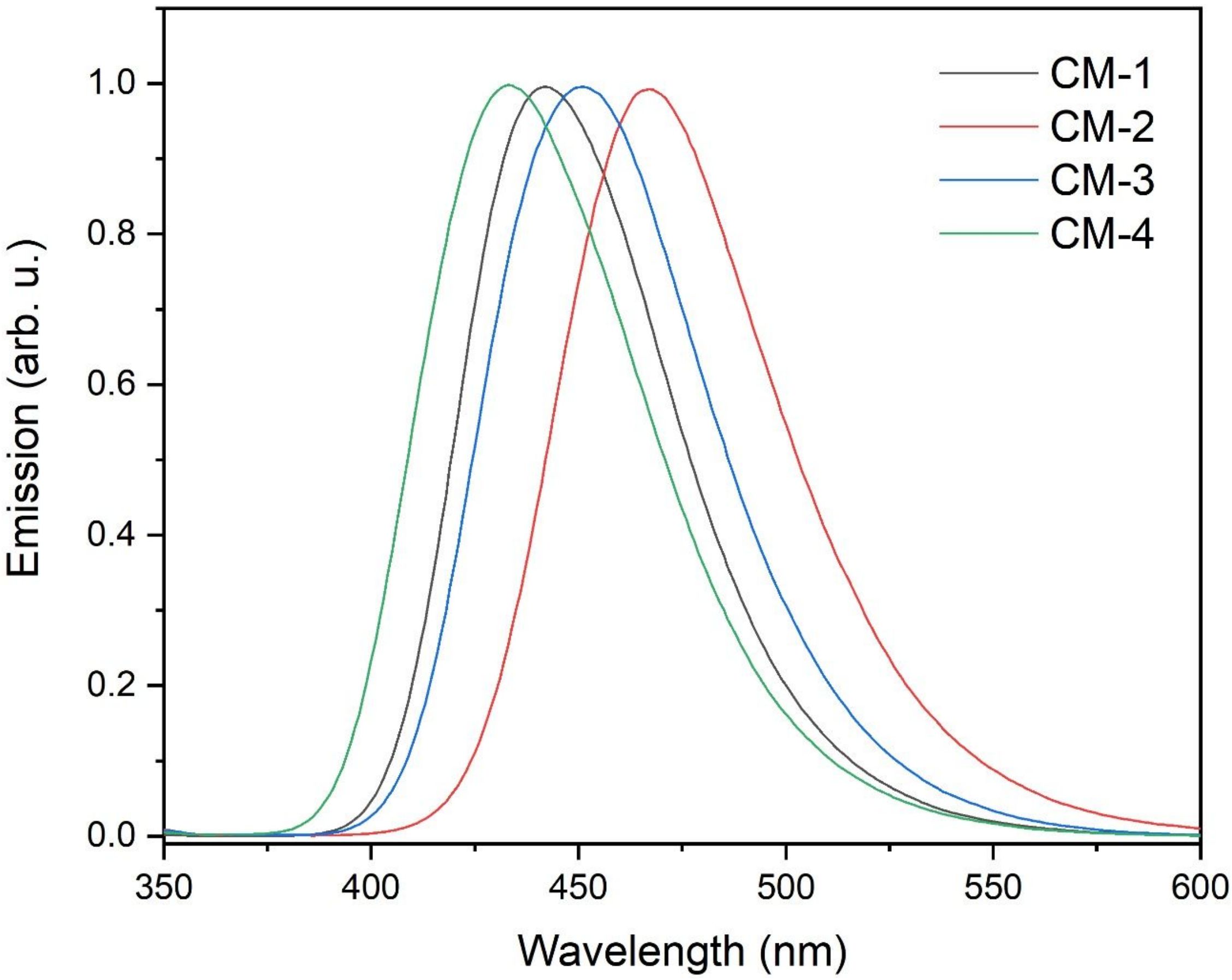

Figure S17. Fluorescence emission spectra of **CM-1** – **CM-4** in ACN, which have been normalized to unity at their maxima.

2.2. Evaluation of the Type I Mechanism

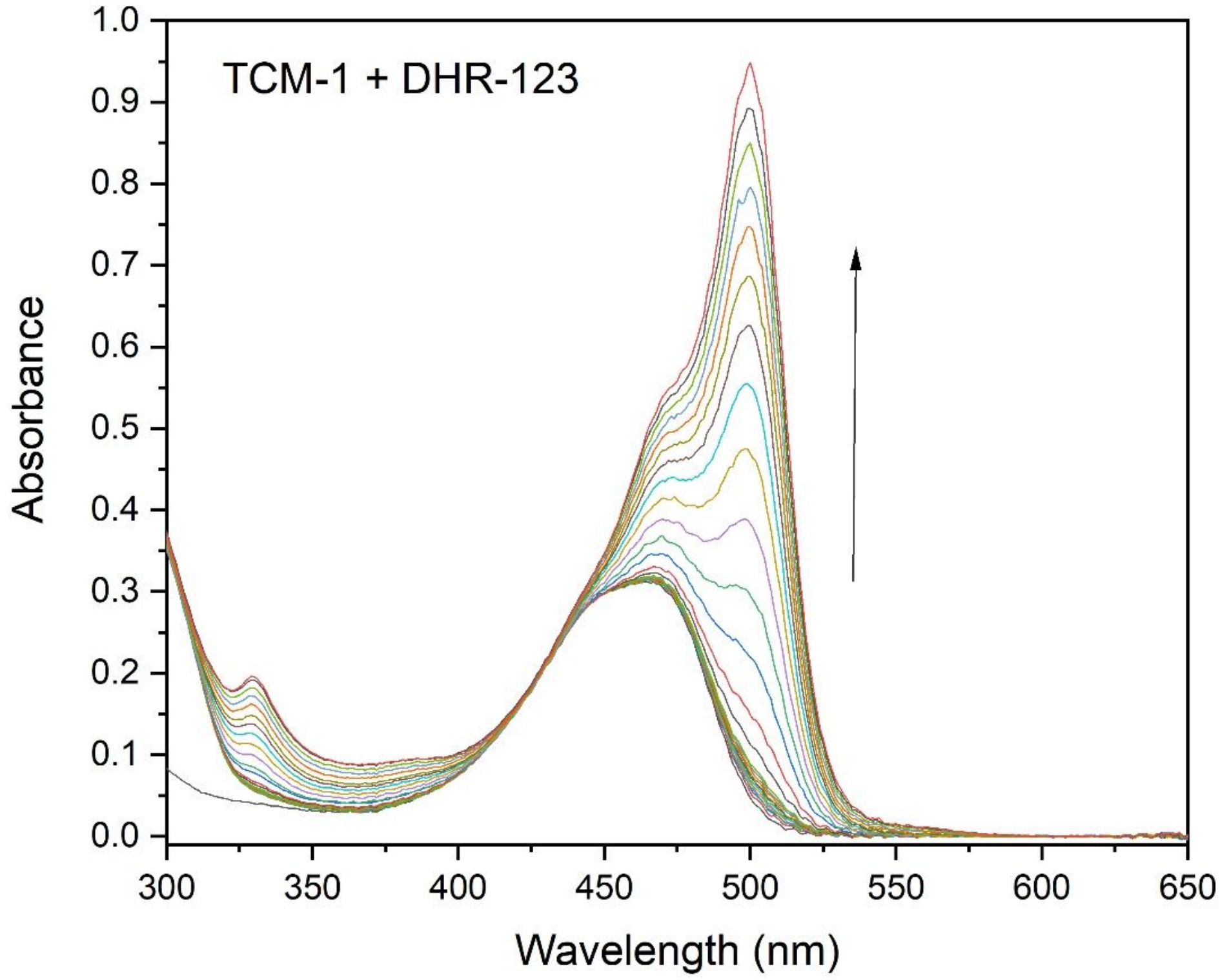

Figure S18. Variation of the absorption spectrum of a mixture of **TCM-1** (c = 1.25 × 10^{-5} M) + DHR-123 (c = 3.75 × 10^{-5} M) in acetonitrile (ACN) upon irradiation with a violet-blue LED (λ_{exc} = 405 nm, power = 71 mW cm^{-2}, irradiation from 0 to 8400 s).

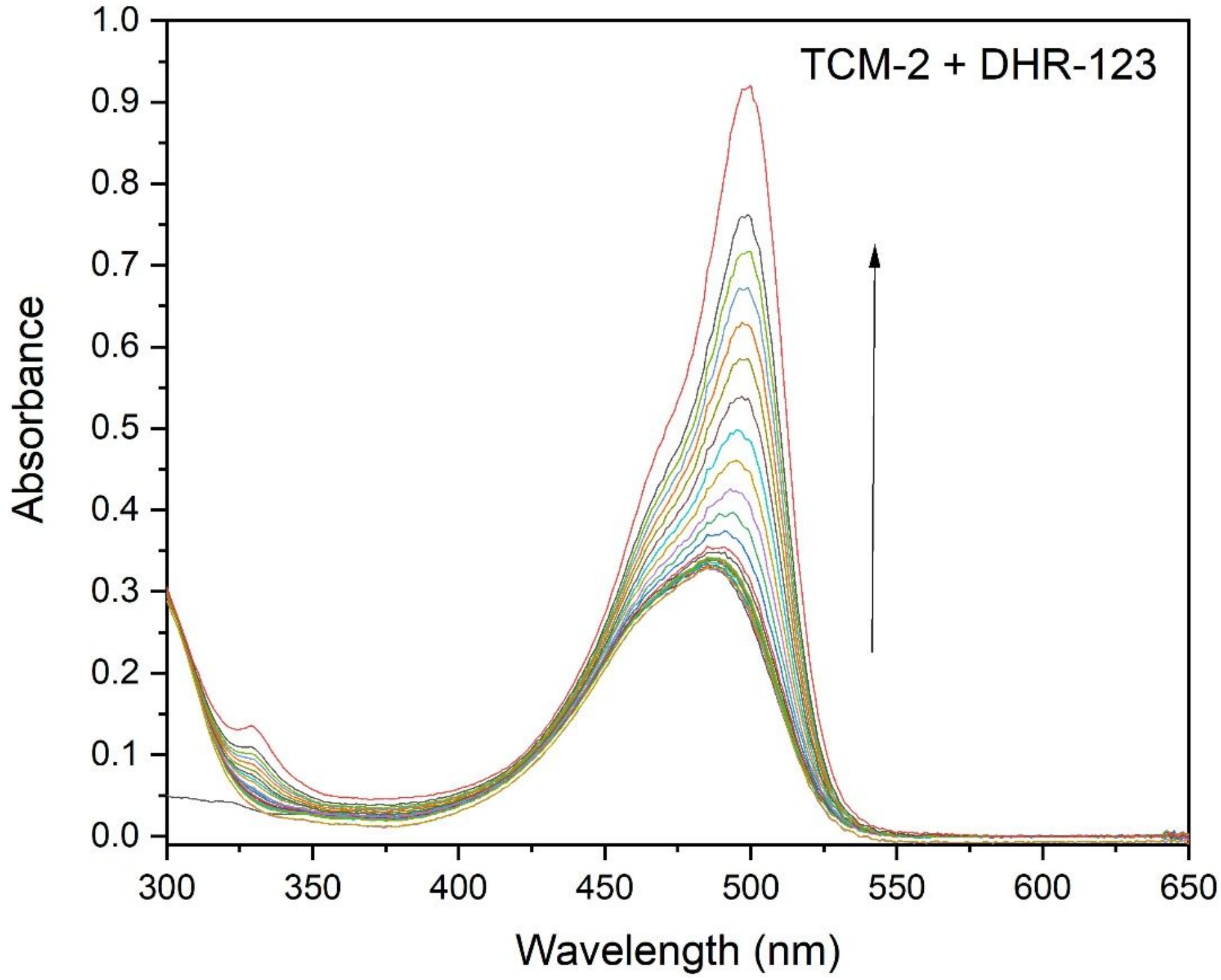

Figure S19. Variation of the absorption spectrum of a mixture of **TCM-2** (c = 1.25 × 10^{-5} M) + DHR-123 (c = 3.75 × 10^{-5} M) in ACN upon irradiation with a violet-blue LED (λ_{exc} = 405 nm, power = 71 mW cm^{-2}, irradiation from 0 to 10800 s).

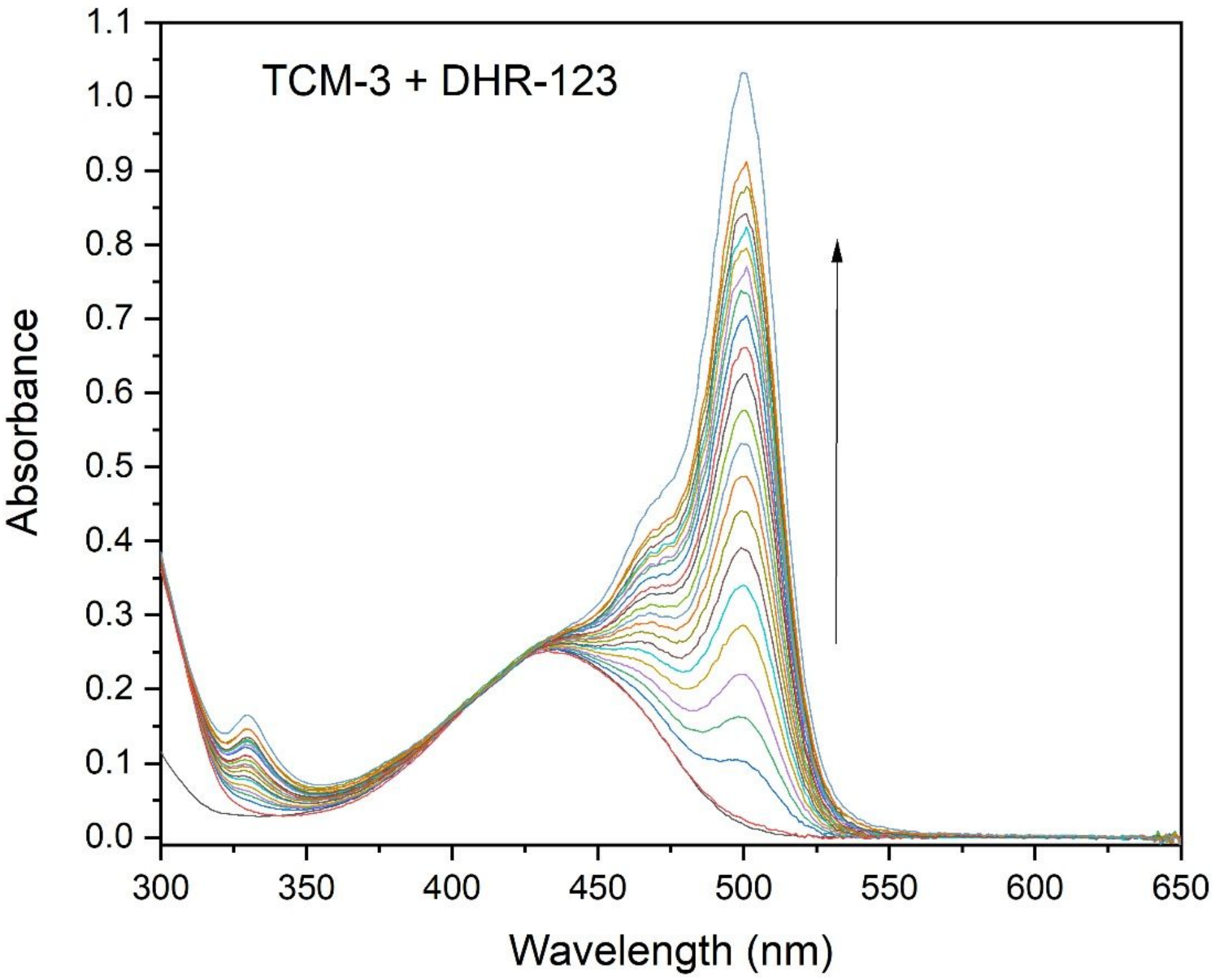

Figure S20. Variation of the absorption spectrum of a mixture of **TCM-3** (c = 1.25 × 10^{-5} M) + DHR-123 (c = 3.75 × 10^{-5} M) in ACN upon irradiation with a violet-blue LED (λ_{exc} = 405 nm, power = 71 mW cm^{-2}, irradiation from 0 to 1500 s).

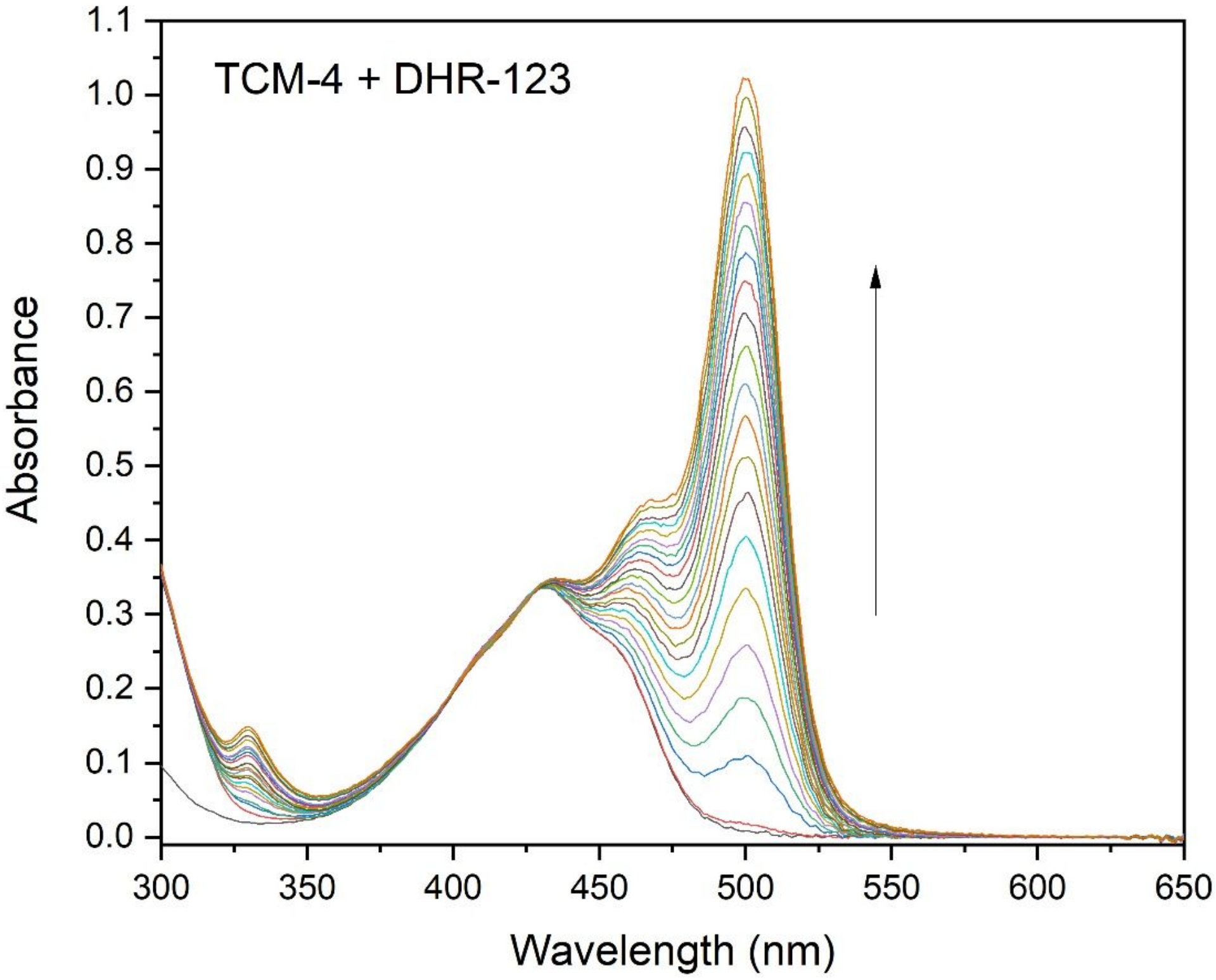

Figure S21. Variation of the absorption spectrum of a mixture of **TCM-4** (c = 1.25 × 10^{-5} M) + DHR-123 (c = 3.75 × 10^{-5} M) in ACN upon irradiation with a violet-blue LED (λ_{exc} = 405 nm, power = 71 mW cm^{-2}, irradiation from 0 to 1200 s).

2.3. Evaluation of the Type II Mechanism

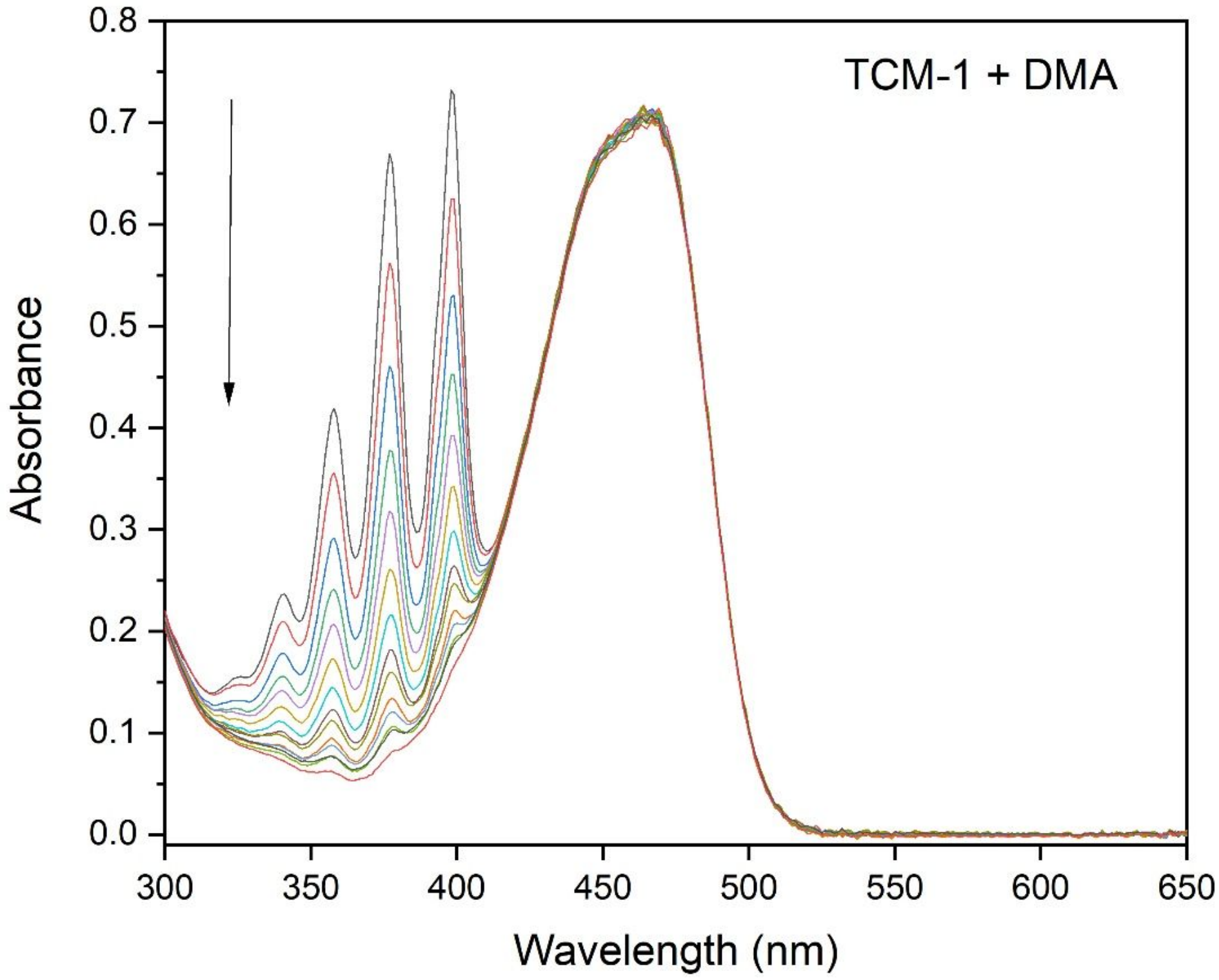

Figure S22. Variation of the absorption spectrum of a mixture of **TCM-1** ($c = 2.5 \times 10^{-5}$ M) + DMA ($c = 7.5 \times 10^{-5}$ M) in ACN upon irradiation with a cw blue laser (λ_{exc} = 450 nm, power = 80 mW cm^{-2}, irradiation from 0 to 900 s).

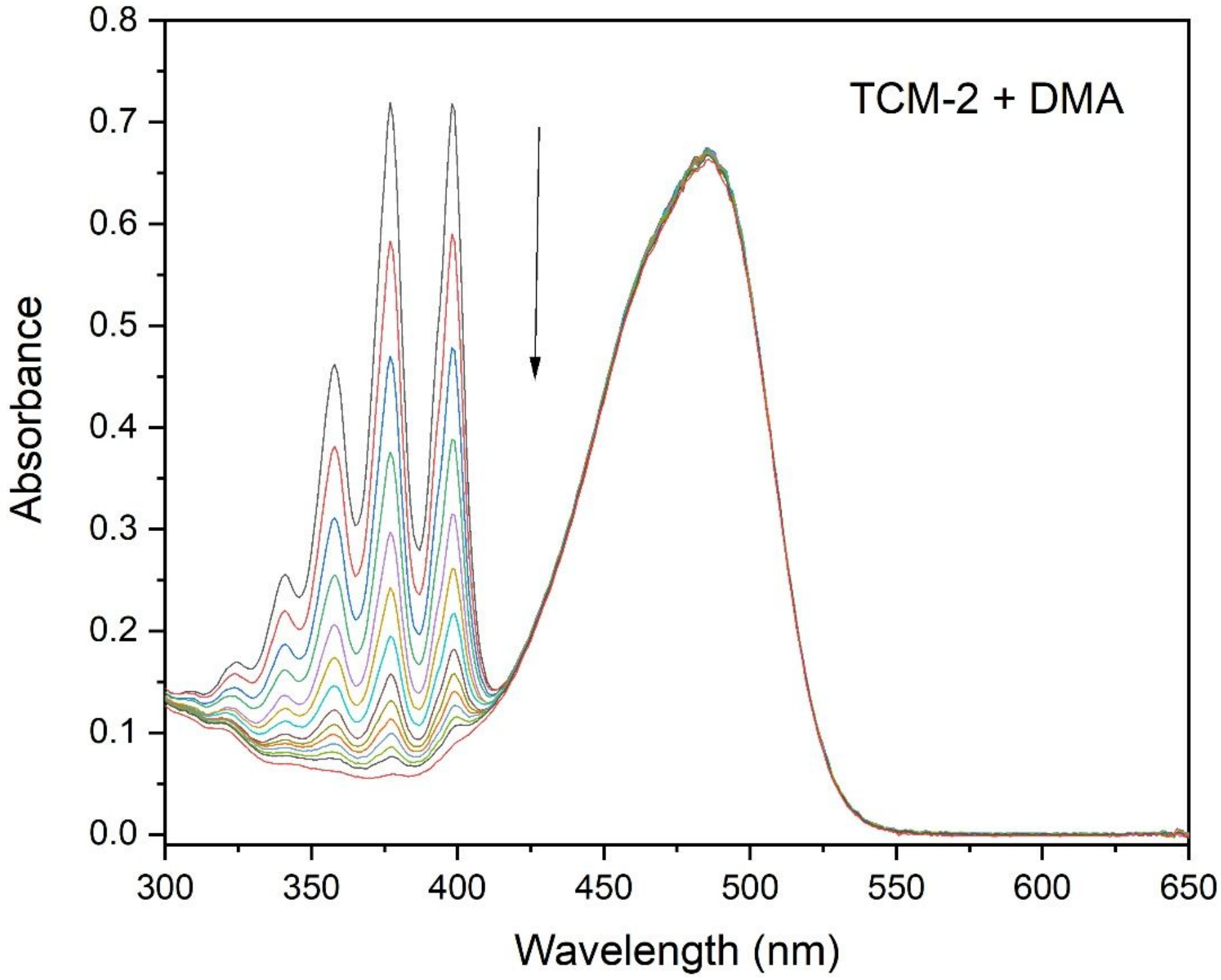

Figure S23. Variation of the absorption spectrum of a mixture of **TCM-2** ($c = 2.5 \times 10^{-5}$ M) + DMA ($c = 7.5 \times 10^{-5}$ M) in ACN upon irradiation with a cw blue laser (λ_{exc} = 450 nm, power = 80 mW cm^{-2}, irradiation from 0 to 900 s).

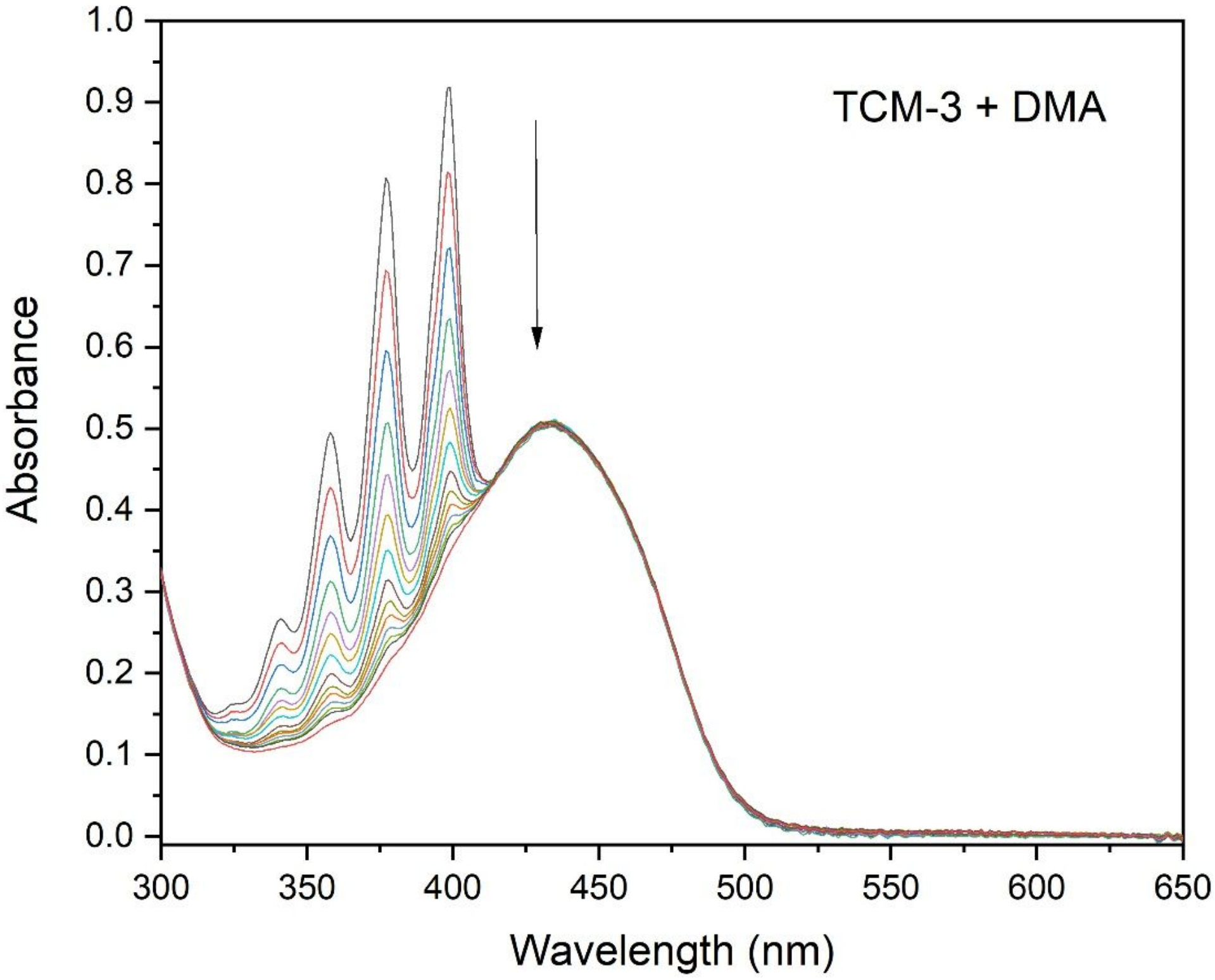

Figure S24. Variation of the absorption spectrum of a mixture of **TCM-3** ($c = 2.5 \times 10^{-5}$ M) + DMA ($c = 7.5 \times 10^{-5}$ M) in ACN upon irradiation with a cw blue laser (λ_{exc} = 450 nm, power = 80 mW cm^{-2}, irradiation from 0 to 900 s).

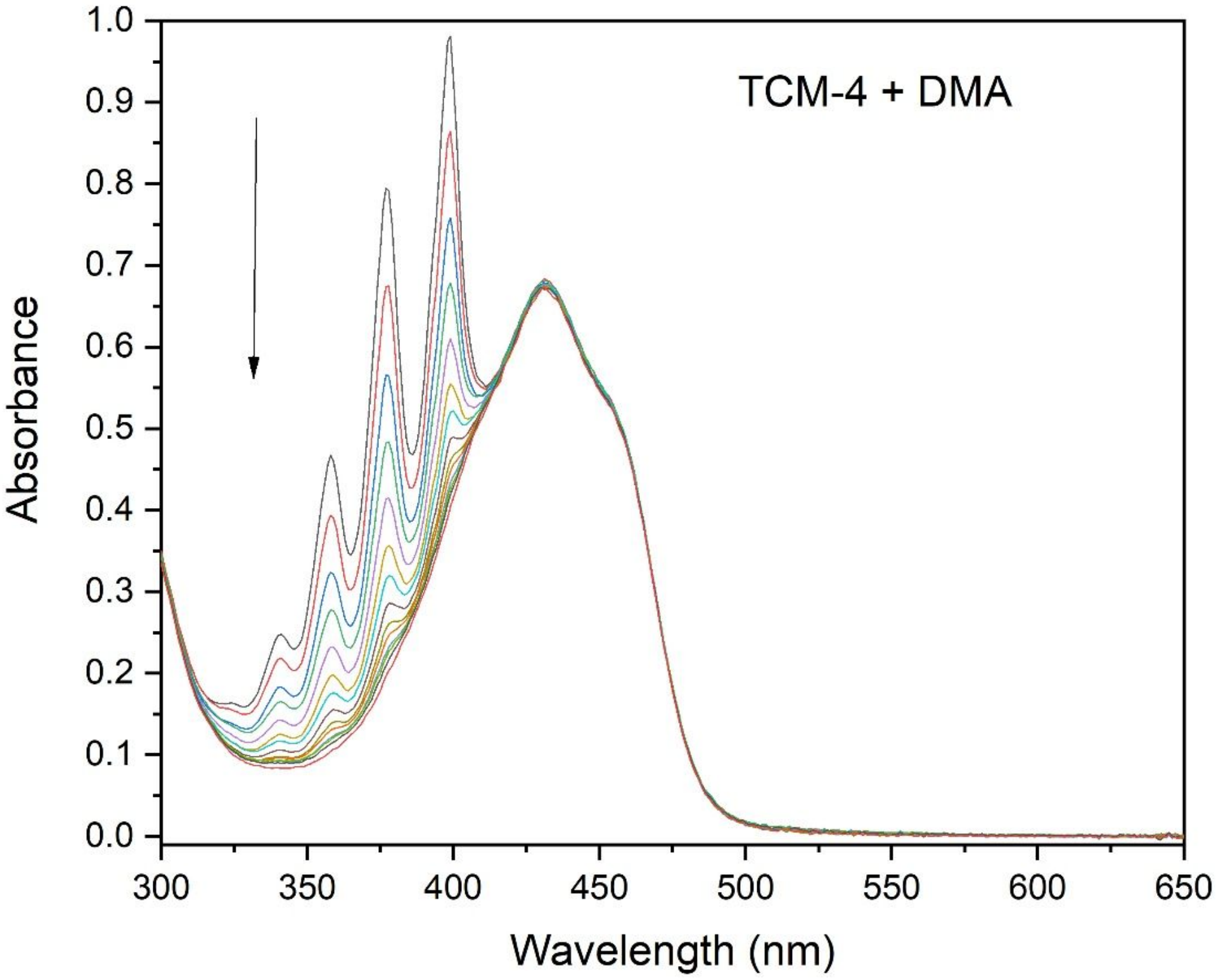

Figure S25. Variation of the absorption spectrum of a mixture of **TCM-4** ($c = 2.5 \times 10^{-5}$ M) + DMA ($c = 7.5 \times 10^{-5}$ M) in ACN upon irradiation with a cw blue laser (λ_{exc} = 450 nm, power = 80 mW cm^{-2}, irradiation from 0 to 900 s).

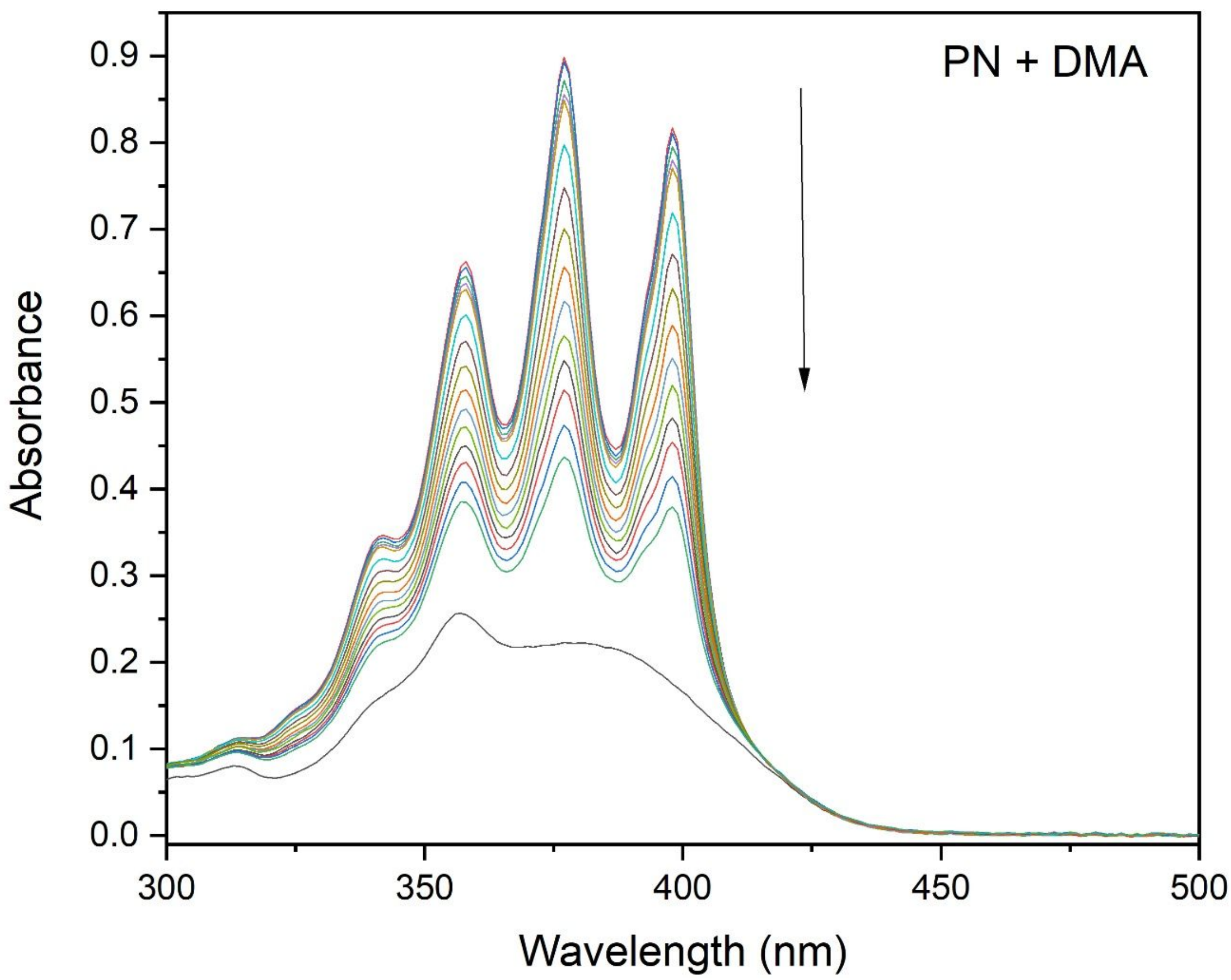

Figure S26. Variation of the absorption spectrum of a mixture of **PN** (c = 2.5 × 10^{-5} M) + DMA (c = 7.5 × 10^{-5} M) in ACN upon irradiation with a cw blue laser (λ_{exc} = 450 nm, power = 80 mW cm^{-2}, irradiation from 0 to 14400 s).

3. X-ray Crystallographic Analysis

TCM-2

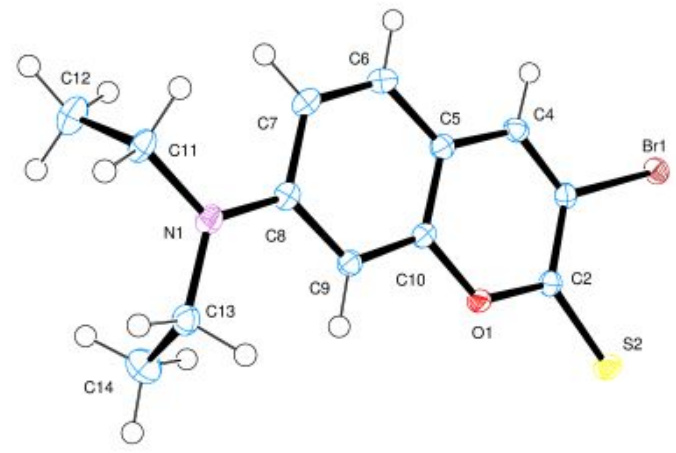

Table S1. Crystal data and structure refinement for **TCM-2**.

Identification code	**TCM-2**
Empirical formula	$C_{13}H_{14}BrNOS$
Formula weight	312.22
Temperature	100(2) K
Wavelength	1.54178 Å
Crystal system	Monoclinic
Space group	P 21/n
Unit cell dimensions	a = 9.2109(4) Å α= 90°.
	b = 11.3878(4) Å β= 105.025(2)°.
	c = 12.7496(6) Å γ = 90°.
Volume	1291.61(10) Å3
Z	4
Density (calculated)	1.606 Mg/m3
Absorption coefficient	5.706 mm-1
F(000)	632
Crystal size	0.310 x 0.240 x 0.110 mm3
Theta range for data collection	5.290 to 77.436°.
Index ranges	-11<=h<=11, -14<=k<=14, -16<=l<=15
Reflections collected	24480
Independent reflections	2674 [R(int) = 0.0431]
Completeness to theta = 67.679°	97.6 %
Refinement method	Full-matrix least-squares on F2
Data / restraints / parameters	2674 / 0 / 156
Goodness-of-fit on F2	1.112
Final R indices [I>2sigma(I)]	R1 = 0.0293, wR2 = 0.0760
R indices (all data)	R1 = 0.0293, wR2 = 0.0760
Largest diff. peak and hole	0.570 and -0.384 e.Å-3

Table S2. Atomic coordinates (× 10^4) and equivalent isotropic displacement parameters ($Å^2$ × 10^3) for **TCM-2**. U(eq) is defined as one-third of the trace of the orthogonalized U^{ij} tensor.

	x	y	z	U(eq)
Br(1)	5788(1)	1323(1)	6787(1)	18(1)
S(2)	7555(1)	3603(1)	8240(1)	19(1)
O(1)	5508(1)	4918(1)	7052(1)	15(1)
N(1)	2296(2)	7918(1)	5137(1)	17(1)
C(2)	6014(2)	3789(2)	7246(1)	14(1)
C(3)	5164(2)	2904(2)	6561(1)	15(1)
C(4)	3953(2)	3173(2)	5733(1)	16(1)
C(5)	3466(2)	4348(2)	5547(1)	15(1)
C(6)	2216(2)	4722(2)	4722(1)	19(1)
C(7)	1813(2)	5880(2)	4602(1)	18(1)
C(8)	2658(2)	6766(2)	5297(1)	15(1)
C(9)	3903(2)	6393(1)	6137(1)	15(1)
C(10)	4268(2)	5217(2)	6235(1)	14(1)
C(11)	1065(2)	8311(2)	4223(2)	19(1)
C(12)	-471(2)	8253(2)	4460(2)	27(1)
C(13)	3127(2)	8823(2)	5863(2)	18(1)
C(14)	2667(2)	8928(2)	6923(2)	26(1)

Table S3. Bond lengths [Å] and angles [°] for **TCM-2**.

Br(1)-C(3)	1.8887(17)
S(2)-C(2)	1.6527(18)
O(1)-C(2)	1.367(2)
O(1)-C(10)	1.375(2)
N(1)-C(8)	1.355(2)
N(1)-C(13)	1.461(2)
N(1)-C(11)	1.469(2)
C(2)-C(3)	1.428(2)
C(3)-C(4)	1.357(2)
C(4)-C(5)	1.412(3)
C(4)-H(4)	0.9500
C(5)-C(10)	1.400(2)
C(5)-C(6)	1.409(2)
C(6)-C(7)	1.367(3)
C(6)-H(6)	0.9500
C(7)-C(8)	1.433(3)
C(7)-H(7)	0.9500
C(8)-C(9)	1.416(2)
C(9)-C(10)	1.378(2)
C(9)-H(9)	0.9500
C(11)-C(12)	1.523(3)
C(11)-H(11A)	0.9900
C(11)-H(11B)	0.9900
C(12)-H(12A)	0.9800
C(12)-H(12B)	0.9800
C(12)-H(12C)	0.9800
C(13)-C(14)	1.523(3)
C(13)-H(13A)	0.9900
C(13)-H(13B)	0.9900
C(14)-H(14A)	0.9800
C(14)-H(14B)	0.9800
C(14)-H(14C)	0.9800
C(2)-O(1)-C(10)	123.21(14)
C(8)-N(1)-C(13)	121.27(15)
C(8)-N(1)-C(11)	121.52(15)
C(13)-N(1)-C(11)	117.21(15)
O(1)-C(2)-C(3)	116.47(15)

O(1)-C(2)-S(2)	116.38(13)
C(3)-C(2)-S(2)	127.15(14)
C(4)-C(3)-C(2)	121.76(16)
C(4)-C(3)-Br(1)	119.34(13)
C(2)-C(3)-Br(1)	118.87(13)
C(3)-C(4)-C(5)	120.40(16)
C(3)-C(4)-H(4)	119.8
C(5)-C(4)-H(4)	119.8
C(10)-C(5)-C(6)	116.78(16)
C(10)-C(5)-C(4)	118.14(16)
C(6)-C(5)-C(4)	125.09(16)
C(7)-C(6)-C(5)	121.38(17)
C(7)-C(6)-H(6)	119.3
C(5)-C(6)-H(6)	119.3
C(6)-C(7)-C(8)	121.45(16)
C(6)-C(7)-H(7)	119.3
C(8)-C(7)-H(7)	119.3
N(1)-C(8)-C(9)	121.58(16)
N(1)-C(8)-C(7)	121.04(16)
C(9)-C(8)-C(7)	117.36(16)
C(10)-C(9)-C(8)	119.41(16)
C(10)-C(9)-H(9)	120.3
C(8)-C(9)-H(9)	120.3
O(1)-C(10)-C(9)	116.42(15)
O(1)-C(10)-C(5)	119.98(15)
C(9)-C(10)-C(5)	123.59(16)
N(1)-C(11)-C(12)	113.30(15)
N(1)-C(11)-H(11A)	108.9
C(12)-C(11)-H(11A)	108.9
N(1)-C(11)-H(11B)	108.9
C(12)-C(11)-H(11B)	108.9
H(11A)-C(11)-H(11B)	107.7
C(11)-C(12)-H(12A)	109.5
C(11)-C(12)-H(12B)	109.5
H(12A)-C(12)-H(12B)	109.5
C(11)-C(12)-H(12C)	109.5
H(12A)-C(12)-H(12C)	109.5
H(12B)-C(12)-H(12C)	109.5
N(1)-C(13)-C(14)	113.39(16)
N(1)-C(13)-H(13A)	108.9

C(14)-C(13)-H(13A)	108.9
N(1)-C(13)-H(13B)	108.9
C(14)-C(13)-H(13B)	108.9
H(13A)-C(13)-H(13B)	107.7
C(13)-C(14)-H(14A)	109.5
C(13)-C(14)-H(14B)	109.5
H(14A)-C(14)-H(14B)	109.5
C(13)-C(14)-H(14C)	109.5
H(14A)-C(14)-H(14C)	109.5
H(14B)-C(14)-H(14C)	109.5

Table S4. Anisotropic displacement parameters ($Å^2 \times 10^3$) for **TCM-2**. The anisotropic displacement factor exponent takes the form: $-2\pi^2[h^2a^{*2}U^{11} + ... + 2 h k a^* b^* U^{12}]$.

	U^{11}	U^{22}	U^{33}	U^{23}	U^{13}	U^{12}
Br(1)	20(1)	12(1)	22(1)	0(1)	5(1)	0(1)
S(2)	17(1)	15(1)	20(1)	2(1)	-2(1)	1(1)
O(1)	14(1)	13(1)	16(1)	0(1)	0(1)	1(1)
N(1)	15(1)	16(1)	18(1)	3(1)	2(1)	1(1)
C(2)	14(1)	14(1)	16(1)	2(1)	5(1)	1(1)
C(3)	14(1)	13(1)	18(1)	0(1)	6(1)	1(1)
C(4)	15(1)	16(1)	17(1)	-3(1)	6(1)	-2(1)
C(5)	15(1)	17(1)	14(1)	0(1)	5(1)	0(1)
C(6)	18(1)	20(1)	17(1)	-2(1)	2(1)	-3(1)
C(7)	15(1)	21(1)	17(1)	2(1)	1(1)	1(1)
C(8)	14(1)	17(1)	15(1)	3(1)	5(1)	0(1)
C(9)	15(1)	13(1)	16(1)	0(1)	3(1)	0(1)
C(10)	13(1)	18(1)	12(1)	1(1)	4(1)	0(1)
C(11)	15(1)	19(1)	21(1)	6(1)	1(1)	3(1)
C(12)	16(1)	31(1)	32(1)	5(1)	5(1)	5(1)
C(13)	17(1)	15(1)	22(1)	2(1)	4(1)	1(1)
C(14)	31(1)	25(1)	24(1)	-3(1)	10(1)	0(1)

Table S5. Hydrogen bonds for **TCM-2** [Å and °].

D-H...A	d(D-H)	d(H...A)	d(D...A)	<(DHA)
C(4)-H(4)...S(2)#1	0.95	2.84	3.7050(18)	151.6
C(13)-H(13B)...S(2)#2	0.99	2.88	3.8514(19)	167.4

Symmetry transformations used to generate equivalent atoms:

#1 x-1/2,-y+1/2,z-1/2 #2 -x+3/2,y+1/2,-z+3/2

TCM-3

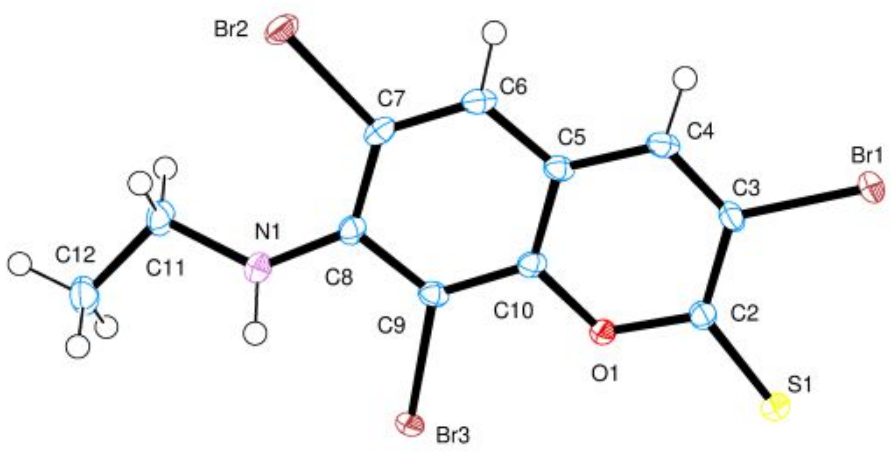

Table S6. Crystal data and structure refinement for **TCM-3**.

Identification code	**TCM-3**
Empirical formula	$C_{11}H_8Br_3NOS$
Formula weight	441.97
Temperature	100(2) K
Wavelength	1.54178 Å
Crystal system	Monoclinic
Space group	P 21/n
Unit cell dimensions	a = 7.6369(7) Å α= 90°.
	b = 13.6565(9) Å β= 93.606(6)°.
	c = 12.2435(10) Å γ = 90°.
Volume	1274.39(18) Å3
Z	4
Density (calculated)	2.304 Mg/m3
Absorption coefficient	13.133 mm-1
F(000)	840
Crystal size	0.300 x 0.070 x 0.030 mm3
Theta range for data collection	6.649 to 72.472°.
Index ranges	-9<=h<=9, -16<=k<=16, -15<=l<=14
Reflections collected	19360
Independent reflections	2430 [R(int) = 0.0489]
Completeness to theta = 67.679°	96.6 %
Refinement method	Full-matrix least-squares on F2
Data / restraints / parameters	2430 / 0 / 156
Goodness-of-fit on F2	1.241
Final R indices [I>2sigma(I)]	R1 = 0.0355, wR2 = 0.0966
R indices (all data)	R1 = 0.0355, wR2 = 0.0966
Extinction coefficient	0.0024(3)
Largest diff. peak and hole	0.841 and -0.704 e.Å-3

Table S7. Atomic coordinates ($\times 10^4$) and equivalent isotropic displacement parameters ($Å^2 \times 10^3$) for **TCM-3**. U(eq) is defined as one-third of the trace of the orthogonalized U^{ij} tensor.

	x	y	z	U(eq)
Br(1)	6179(1)	4231(1)	9047(1)	19(1)
Br(2)	9597(1)	3431(1)	3024(1)	20(1)
Br(3)	6671(1)	7129(1)	4338(1)	18(1)
S(1)	5240(1)	6504(1)	8183(1)	19(1)
O(1)	6208(3)	5971(2)	6306(2)	14(1)
N(1)	8345(4)	5743(2)	2768(3)	21(1)
C(2)	5999(4)	5679(2)	7360(3)	15(1)
C(3)	6464(5)	4679(3)	7615(3)	16(1)
C(4)	7132(4)	4071(2)	6873(3)	16(1)
C(5)	7397(4)	4420(2)	5805(3)	15(1)
C(6)	8154(4)	3873(3)	4979(3)	17(1)
C(7)	8452(4)	4277(3)	3985(3)	16(1)
C(8)	8021(4)	5263(2)	3710(3)	15(1)
C(9)	7247(4)	5797(2)	4548(3)	15(1)
C(10)	6944(4)	5388(2)	5551(3)	14(1)
C(11)	8741(5)	5377(3)	1687(3)	20(1)
C(12)	8739(5)	6234(3)	912(3)	24(1)

Table S8. Bond lengths [Å] and angles [°] for **TCM-3**.

Br(1)-C(3)	1.883(3)
Br(2)-C(7)	1.902(3)
Br(3)-C(9)	1.885(3)
S(1)-C(2)	1.641(3)
O(1)-C(10)	1.367(4)
O(1)-C(2)	1.370(4)
N(1)-C(8)	1.362(5)
N(1)-C(11)	1.465(4)
N(1)-H(1)	0.8800
C(2)-C(3)	1.441(5)
C(3)-C(4)	1.354(5)
C(4)-C(5)	1.418(5)
C(4)-H(4)	0.9500
C(5)-C(10)	1.396(5)
C(5)-C(6)	1.410(5)
C(6)-C(7)	1.367(5)
C(6)-H(6)	0.9500
C(7)-C(8)	1.422(5)
C(8)-C(9)	1.418(5)
C(9)-C(10)	1.382(5)
C(11)-C(12)	1.506(5)
C(11)-H(11A)	0.9900
C(11)-H(11B)	0.9900
C(12)-H(12A)	0.9800
C(12)-H(12B)	0.9800
C(12)-H(12C)	0.9800
C(10)-O(1)-C(2)	123.0(3)
C(8)-N(1)-C(11)	131.3(3)
C(8)-N(1)-H(1)	114.4
C(11)-N(1)-H(1)	114.4
O(1)-C(2)-C(3)	116.0(3)
O(1)-C(2)-S(1)	116.5(2)
C(3)-C(2)-S(1)	127.5(3)
C(4)-C(3)-C(2)	122.3(3)
C(4)-C(3)-Br(1)	119.8(3)
C(2)-C(3)-Br(1)	117.9(3)
C(3)-C(4)-C(5)	119.6(3)
C(3)-C(4)-H(4)	120.2
C(5)-C(4)-H(4)	120.2
C(10)-C(5)-C(6)	116.6(3)
C(10)-C(5)-C(4)	118.4(3)

C(6)-C(5)-C(4)	124.9(3)
C(7)-C(6)-C(5)	121.6(3)
C(7)-C(6)-H(6)	119.2
C(5)-C(6)-H(6)	119.2
C(6)-C(7)-C(8)	122.9(3)
C(6)-C(7)-Br(2)	114.5(3)
C(8)-C(7)-Br(2)	122.5(3)
N(1)-C(8)-C(9)	118.1(3)
N(1)-C(8)-C(7)	127.2(3)
C(9)-C(8)-C(7)	114.6(3)
C(10)-C(9)-C(8)	122.4(3)
C(10)-C(9)-Br(3)	117.5(3)
C(8)-C(9)-Br(3)	120.1(3)
O(1)-C(10)-C(9)	117.6(3)
O(1)-C(10)-C(5)	120.5(3)
C(9)-C(10)-C(5)	121.9(3)
N(1)-C(11)-C(12)	108.2(3)
N(1)-C(11)-H(11A)	110.1
C(12)-C(11)-H(11A)	110.1
N(1)-C(11)-H(11B)	110.1
C(12)-C(11)-H(11B)	110.1
H(11A)-C(11)-H(11B)	108.4
C(11)-C(12)-H(12A)	109.5
C(11)-C(12)-H(12B)	109.5
H(12A)-C(12)-H(12B)	109.5
C(11)-C(12)-H(12C)	109.5
H(12A)-C(12)-H(12C)	109.5
H(12B)-C(12)-H(12C)	109.5

Table S9. Anisotropic displacement parameters ($Å^2 \times 10^3$) for **TCM-3**. The anisotropic displacement factor exponent takes the form: $-2\pi^2[h^2a^{*2}U^{11} + ... + 2 h k a^* b^* U^{12}]$.

	U^{11}	U^{22}	U^{33}	U^{23}	U^{13}	U^{12}
Br(1)	26(1)	17(1)	13(1)	4(1)	1(1)	0(1)
Br(2)	26(1)	17(1)	16(1)	-6(1)	1(1)	3(1)
Br(3)	29(1)	10(1)	15(1)	2(1)	2(1)	0(1)
S(1)	28(1)	14(1)	16(1)	-1(1)	4(1)	2(1)
O(1)	19(1)	12(1)	12(1)	0(1)	1(1)	1(1)
N(1)	32(2)	17(2)	13(1)	-2(1)	4(1)	0(1)
C(2)	17(2)	15(2)	13(2)	0(1)	0(1)	-3(1)
C(3)	22(2)	15(2)	12(2)	4(1)	0(1)	-1(1)
C(4)	19(2)	11(2)	18(2)	1(1)	-5(1)	-1(1)
C(5)	16(2)	12(2)	15(2)	1(1)	-4(1)	0(1)
C(6)	19(2)	12(2)	19(2)	-2(1)	-5(1)	2(1)
C(7)	17(2)	16(2)	16(2)	-6(1)	1(1)	0(1)
C(8)	16(2)	17(2)	11(2)	-3(1)	-2(1)	-3(1)
C(9)	16(2)	12(2)	16(2)	1(1)	-3(1)	1(1)
C(10)	14(2)	13(2)	14(2)	-2(1)	-3(1)	-2(1)
C(11)	23(2)	24(2)	11(2)	-2(1)	-1(1)	-2(1)
C(12)	30(2)	28(2)	15(2)	1(2)	3(2)	-5(2)

Table S10. Hydrogen bonds for **TCM-3** [Å and °].

D-H...A	d(D-H)	d(H...A)	d(D...A)	<(DHA)
N(1)-H(1)...Br(3)	0.88	2.52	3.036(3)	118.2
C(12)-H(12C)...Br(1)#1	0.98	3.08	3.812(4)	133.0

Symmetry transformations used to generate equivalent atoms:

#1 -x+1,-y+1,-z+1

TCM-4

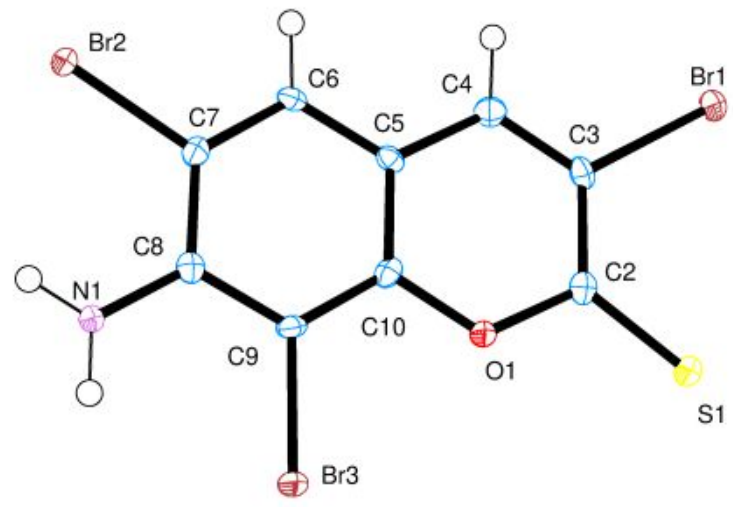

Table S11. Crystal data and structure refinement for **TCM-4**.

Identification code	**TCM-4**	
Empirical formula	**$C_9H_4Br_3NOS$**	
Formula weight	413.89	
Temperature	100(2) K	
Wavelength	0.71073 Å	
Crystal system	Monoclinic	
Space group	P 21/c	
Unit cell dimensions	a = 11.8638(5) Å	α= 90°.
	b = 13.6002(5) Å	β= 99.4480(10)°.
	c = 6.8360(3) Å	γ = 90°.
Volume	1088.03(8) $Å^3$	
Z	4	
Density (calculated)	2.527 Mg/m^3	
Absorption coefficient	11.283 mm^{-1}	
F(000)	776	
Crystal size	0.180 x 0.170 x 0.020 mm^3	
Theta range for data collection	2.296 to 27.528°.	
Index ranges	-15<=h<=15, -17<=k<=17, -8<=l<=8	
Reflections collected	41992	
Independent reflections	2505 [R(int) = 0.0894]	
Completeness to theta = 25.242°	99.9 %	
Refinement method	Full-matrix least-squares on F^2	
Data / restraints / parameters	2505 / 0 / 136	
Goodness-of-fit on F^2	1.120	
Final R indices [I>2sigma(I)]	R1 = 0.0273, wR2 = 0.0578	
R indices (all data)	R1 = 0.0385, wR2 = 0.0639	
Largest diff. peak and hole	0.624 and -0.536 e.$Å^{-3}$	

Table S12. Atomic coordinates ($\times 10^4$) and equivalent isotropic displacement parameters ($Å^2 \times 10^3$) for **TCM-4**. U(eq) is defined as one-third of the trace of the orthogonalized U^{ij} tensor.

	x	y	z	U(eq)
Br(1)	10195(1)	8769(1)	5827(1)	18(1)
Br(2)	7587(1)	3306(1)	3906(1)	20(1)
Br(3)	4724(1)	6690(1)	2392(1)	15(1)
S(1)	7524(1)	9527(1)	4206(1)	15(1)
O(1)	6943(2)	7695(2)	3912(4)	13(1)
N(1)	5355(3)	4487(2)	2668(5)	16(1)
C(2)	7834(3)	8354(3)	4457(5)	13(1)
C(3)	8928(3)	7925(3)	5157(5)	13(1)
C(4)	9086(3)	6938(3)	5302(5)	14(1)
C(5)	8168(3)	6289(3)	4676(5)	11(1)
C(6)	8280(3)	5262(3)	4679(5)	12(1)
C(7)	7350(3)	4686(3)	3995(5)	13(1)
C(8)	6255(3)	5078(3)	3318(5)	12(1)
C(9)	6154(3)	6107(3)	3343(5)	12(1)
C(10)	7092(3)	6693(3)	3981(5)	12(1)

Table S13. Bond lengths [Å] and angles [°] for **TCM-4**.

Br(1)-C(3)	1.886(4)
Br(2)-C(7)	1.901(4)
Br(3)-C(9)	1.888(4)
S(1)-C(2)	1.639(4)
O(1)-C(10)	1.374(4)
O(1)-C(2)	1.389(4)
N(1)-C(8)	1.352(5)
C(2)-C(3)	1.432(5)
C(3)-C(4)	1.357(5)
C(4)-C(5)	1.413(5)
C(5)-C(10)	1.399(5)
C(5)-C(6)	1.402(5)
C(6)-C(7)	1.372(5)
C(7)-C(8)	1.410(5)
C(8)-C(9)	1.406(5)
C(9)-C(10)	1.380(5)
C(10)-O(1)-C(2)	122.9(3)
O(1)-C(2)-C(3)	115.7(3)
O(1)-C(2)-S(1)	116.9(3)
C(3)-C(2)-S(1)	127.4(3)
C(4)-C(3)-C(2)	122.3(3)
C(4)-C(3)-Br(1)	119.3(3)
C(2)-C(3)-Br(1)	118.4(3)
C(3)-C(4)-C(5)	120.4(4)
C(10)-C(5)-C(6)	118.2(3)
C(10)-C(5)-C(4)	118.2(3)
C(6)-C(5)-C(4)	123.6(3)
C(7)-C(6)-C(5)	119.8(3)
C(6)-C(7)-C(8)	122.9(3)
C(6)-C(7)-Br(2)	117.4(3)
C(8)-C(7)-Br(2)	119.7(3)
N(1)-C(8)-C(9)	122.1(3)
N(1)-C(8)-C(7)	121.2(3)
C(9)-C(8)-C(7)	116.6(3)
C(10)-C(9)-C(8)	120.8(3)
C(10)-C(9)-Br(3)	119.9(3)
C(8)-C(9)-Br(3)	119.2(3)

O(1)-C(10)-C(9)	118.0(3)
O(1)-C(10)-C(5)	120.4(3)
C(9)-C(10)-C(5)	121.6(3)

Table S14. Anisotropic displacement parameters ($Å^2 \times 10^3$) for **TCM-4**. The anisotropic displacement factor exponent takes the form: $-2\pi^2[h^2a^{*2}U^{11} + ... + 2 h k a^* b^* U^{12}]$.

	U^{11}	U^{22}	U^{33}	U^{23}	U^{13}	U^{12}
Br(1)	13(1)	12(1)	27(1)	-2(1)	-1(1)	-3(1)
Br(2)	12(1)	9(1)	37(1)	-1(1)	1(1)	0(1)
Br(3)	11(1)	12(1)	22(1)	0(1)	-2(1)	2(1)
S(1)	15(1)	11(1)	19(1)	0(1)	2(1)	0(1)
O(1)	11(1)	11(1)	16(1)	1(1)	0(1)	0(1)
N(1)	10(2)	10(1)	26(2)	-1(1)	-1(1)	-1(1)
C(2)	16(2)	13(2)	9(2)	0(1)	4(1)	-2(1)
C(3)	13(2)	14(2)	13(2)	-1(1)	1(2)	-5(1)
C(4)	14(2)	16(2)	12(2)	0(1)	0(1)	1(1)
C(5)	9(2)	14(2)	9(2)	2(1)	1(1)	-1(1)
C(6)	7(2)	15(2)	15(2)	0(1)	1(1)	1(1)
C(7)	15(2)	10(2)	15(2)	1(1)	2(2)	0(1)
C(8)	14(2)	13(2)	11(2)	-1(1)	4(1)	-2(1)
C(9)	10(2)	12(2)	14(2)	1(1)	3(1)	4(1)
C(10)	16(2)	10(2)	11(2)	-1(1)	6(1)	1(1)

Table S15. Hydrogen bonds for **TCM-4** [Å and °].

D-H...A	d(D-H)	d(H...A)	d(D...A)	<(DHA)
N(1)-H(1A)...Br(3)	0.88	2.65	3.087(3)	111.9
N(1)-H(1A)...S(1)#1	0.88	2.65	3.446(3)	150.1
N(1)-H(1B)...Br(2)	0.88	2.65	3.094(3)	112.7
N(1)-H(1B)...Br(3)#1	0.88	2.94	3.805(3)	169.2
C(4)-H(4)...Br(2)#2	0.95	3.05	3.910(4)	150.6

Symmetry transformations used to generate equivalent atoms:

#1 -x+1,y-1/2,-z+1/2   #2 -x+2,-y+1,-z+1
